# Simulations of Functional Motions of Super Large Biomolecules with a Mixed-Resolution Model

**DOI:** 10.1021/acs.jctc.3c01046

**Published:** 2024-02-20

**Authors:** Shu Li, Bohua Wu, Yun Lyna Luo, Wei Han

**Affiliations:** † Centre for Artificial Intelligence Driven Drug Discovery, Faculty of Applied Sciences, Macao Polytechnic University, Macao 999078, China; ‡ State Key Laboratory of Chemical Oncogenomics, Guangdong Provincial Key Laboratory of Chemical Genomics, School of Chemical Biology and Biotechnology, Peking University Shenzhen Graduate School, Shenzhen 518055, China; § Department of Chemistry, Faculty of Science, Hong Kong Baptist University, Hong Kong SAR 999077, China; ∥ Shenzhen Bay Laboratory, Institute of Chemical Biology, Shenzhen 518132, China; ⊥ Department of Biotechnology and Pharmaceutical Sciences, Western University of Health Sciences, Pomona, California 91766, United States

## Abstract

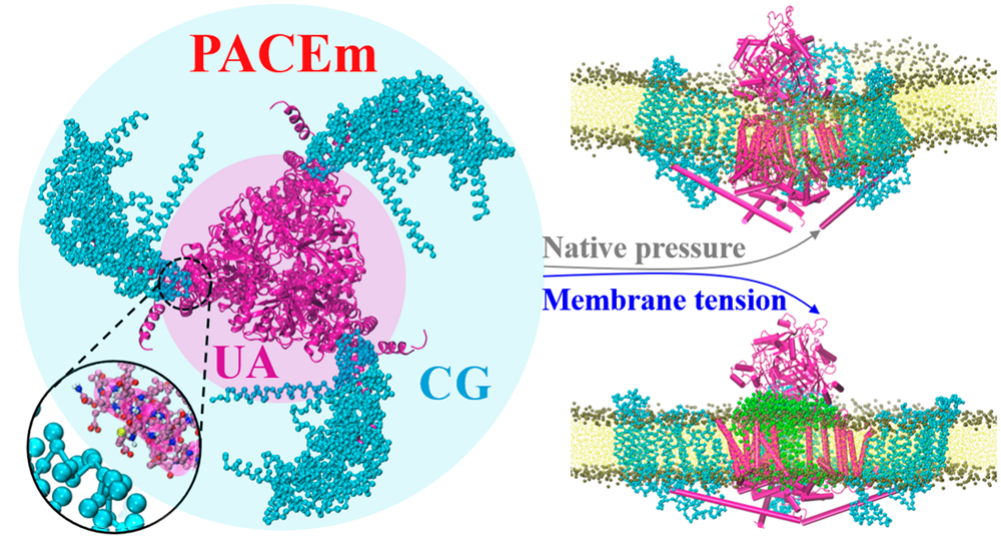

Many large protein machines function through an interplay between large-scale movements and intricate conformational changes. Understanding functional motions of these proteins through simulations becomes challenging for both all-atom and coarse-grained (CG) modeling techniques because neither approach alone can readily capture the full details of these motions. In this study, we develop a multiscale model by employing the popular MARTINI CG model to represent a heterogeneous environment and structurally stable proteins and using the united-atom (UA) model PACE to describe proteins undergoing subtle conformational changes. PACE was previously developed to be compatible with the MARTINI solvent and membrane. Here, we couple the protein descriptions of the two models by directly mixing UA and CG interaction parameters to greatly simplify parameter determination. Through extensive validations with diverse protein systems in solution or membrane, we demonstrate that only additional parameter rescaling is needed to enable the resulting model to recover the stability of native structures of proteins under mixed representation. Moreover, we identify the optimal scaling factors that can be applied to various protein systems, rendering the model potentially transferable. To further demonstrate its applicability for realistic systems, we apply the model to a mechanosensitive ion channel Piezo1 that has peripheral arms for sensing membrane tension and a central pore for ion conductance. The model can reproduce the coupling between Piezo1’s large-scale arm movement and subtle pore opening in response to membrane stress while consuming much less computational costs than all-atom models. Therefore, our model shows promise for studying functional motions of large protein machines.

## 1 Introduction

Proteins play a crucial role in various biological functions.^1^ In particular, large protein machines, comprised of multiple subunits or domains of hundreds or even thousands of amino acids, are required for complex tasks such as sensory perception, mechanical sensor, and cell movement.^2−4^ Understanding how these protein machines fulfill their functions is of central importance to biological research and relies on the knowledge of structures and dynamics of proteins. Although recent advancements in cryo-electron microscopy (cryo-EM) and X-ray techniques have enabled structural characterization of large and complex protein structures,^5,6^ their conformational dynamics remain crucial for understanding protein functions but missing. There is still a lack of experimental methods that can resolve dynamics of complex protein machines with sufficient details. This presents an exciting opportunity for molecular dynamics (MD) simulations that may provide invaluable insights complementing experiment observations.^7,8^

Conventional all-atom (AA) MD simulations are arguably the most accurate and straightforward way of modeling proteins, but their computation cost is extraordinarily high for complex protein machines because of the need to model millions of interacting atomistic sites.^9,10^ Coarse-grained (CG) simulations use single sites to represent all the atoms of a chemical group, a residue, or even a domain, allowing simulations to last longer^11−15^ and access extremely large systems.^16^ Although AA and CG models have been used in the study of a wide range of biomolecular processes, neither approach alone is applicable for the study of complex protein machines whose functional motions involve a synergistic interplay between large scale domain movements and precise structural changes of a subset of atoms, as was exemplified by numerous large mechanosensitive protein systems^2,3,17−19^ and motor protein systems.^4,20,21^ A possible solution to this problem is to include both AA and CG representations in the same simulations and represent each region of the system with a specific resolution based on the nature of motions of that region.

The parametrization of a model with mixed resolutions is challenging due to the requirement for models of high quality at both resolutions and an appropriate strategy to couple the models across resolutions. To this end, a general approach is the multiscale coarse-graining (MSCG) scheme used first by Shi et al.^22^ to develop a mixed AA/CG model for simulating the gramicidin A (gA) ion channel and by Shelley et al. to study peptide aggregation.^23^ The AA-AA, CG-CG, and AA-CG interaction parameters for various components of systems can all be obtained rigorously by using the force-matching techniques to reproduce free energy surface obtained from AA simulations of the same systems. However, the success of this approach relies on a sufficient sampling of underlying AA systems which is difficult for complex protein machines. Also, the model obtained is system-dependent and may not be transferable.

Rzepiela et al. proposed a scheme to combine the GROMOS united-atom (UA) model with the MARTINI CG models for water and membrane,^24,25^ the latter model having been commonly used in large scale simulations of biomolecules in membrane environments. In order to avoid cumbersome parametrization across resolutions, additional virtual CG sites were introduced as proxies of AA sites. These virtual sites rather than AA sites were used to evaluate the interactions with the CG sites, and their interactions are described using the parameters from MARTINI.^24^ Zacharias et al.^26^ developed also a similar approach based on virtual CG sites using a knowledge-based potential for these CG sites. Another approach by Xia and co-workers^27^ employed elastic networks to model interactions with virtual sites. Nonetheless, it remains unclear whether the AA models that are optimized for an atomistic water model could be transferable for a CG water environment. Incidentally, as was found by Sokkar et al.,^28^ the coupling scheme proposed by Rzepiela et al.^24^ may result in too strong AA-CG interactions that cause unfolding of AA proteins in simulations. A systematic reparametrization of the interactions involving virtual sites is thus necessary and may be attainable, as was shown by Ge et al. in their recent model development for peptide self-assembly using machine learning techniques.^29^

In a different type of approaches, AA protein sites were allowed to interact with polarizable CG water sites through electrostatic and van der Waals (vdW) interactions. The parameters for these interactions were generated from those of the AA and CG sites using a set of combining rules^30,31^ but could be rescaled^32,33^ or even systematically optimized^34,35^ to reproduce solvation free energies of small molecules or their association free energy profiles.^36^ Due to the improved description of interactions of AA protein sites with CG water, these models can be used to reproduce membrane permeability of small solutes^30^ and maintain folded structures of proteins in water^33,34^ and membrane.^37^ However, these models still need a CG presentation of proteins and a proper coupling scheme to be applicable for complex protein simulations in which both AA and CG representations of proteins are present.

In an interesting work of Predeus et al.,^38^ a mixed representation of protein was implemented. Their model coupled the CHARMM AA force field with an intermediate-resolution CG model called PRIMO^39^ that was parametrized to be compatible with CHARMM in terms of energetics of model peptides and proteins. Moreover, a unique resolution-switching scheme was introduced to mix the two representations within the same molecule. The mixed AA/CG model was employed to simulate conformational preference of peptides (at an AA level) in a crowded environment represented by CG proteins.^38^ More recently, this approach is further developed to allow for a mixed AA/CG representation within the same protein molecules.^40^ Nevertheless, as the mixed model represents implicitly the surrounding environment including water and membrane, its applicability for membrane-related simulations might be limited, especially when the change of membrane structures is the topic of interest.

It is worth mentioning that another approach called the Multi-Scale Enhanced Sampling (MSES) couples AA models with low-resolution models at a Hamiltonian level.^41−43^ This method was developed to enhance the sampling of the functional transitions of proteins between their different conformational states. The transitions are sampled with the AA models but guided by the low-resolution models. A key advantage of this approach is that no physical interactions across resolutions need to be considered except for the restraining forces generated by low-resolution potentials to restrict AA sampling space. The construction of these low-resolution models, however, requires the prior structural knowledge on all the key conformational states associated with the functional transitions, which is not always possible. Also, the need for this approach to represent the entire systems at the AA level may still render simulations of very large biomolecular systems computationally demanding.

In this study, we seek to develop an efficient mixed resolution model that can be readily applied to study large complex protein machines in water and membrane. We couple a UA force field called PACE developed in our group with the MARTINI CG model.^44−48^ Unlike most of the AA models, the PACE model was designed to be compatible with the MARTINI CG water and lipid model such that the mixed model can predict solvation free energies of small solutes^47^ and their transfer free energies from water to membrane.^49^ Moreover, the UA protein parts of PACE retain key features of interactions like hydrogen bonding (HB) and π–π stacking and were calibrated to reproduce the statistical distribution of peptide conformers.^44,45^ As a result, several model proteins could retain their native structures during simulations with PACE,^46,49^ and more importantly, ab initio folding of multiple small proteins was also observed in the simulations.^44,46,48^ Thus, a combination of the PACE/MARTINI scheme is a feasible route to a multiscale modeling of complex protein systems. In such systems, PACE can be used to probe the conformational transitions of key protein regions, while the rest of the parts of proteins are deemed as surrounding protein environments that can be modeled efficiently using MARTINI coupled with an elastic network potential like ELNEDYN that can maintain the structural stability of these regions.^50^

To this end, we need to establish the coupling between the PACE model and the MARTINI CG protein model. The main task is to parametrize the nonbonded interactions between PACE UA and MARTINI CG protein sites. In PACE, the nonbonded interactions between UA protein sites and MARTINI CG water sites were both parametrized and validated by reproducing experimental hydration free energies of many small organic compounds^47^ since this quantity is directly related to the strength of protein–solvent interactions. This strategy is practical because of the availability of a large amount of experimental data, but it cannot be applied to the UA/CG interaction parametrization for protein sites due to a significantly more parameters that need to be determined and the lack of experimental quantities analogous to hydration free energies. To reduce the complexity of the parametrization procedure, we follow previous studies^40,51^ and directly mix the nonbonded parameters for PACE UA and MARTINI CG protein sites using the combination rules. Additional scaling factors are introduced to adjust the mixed nonbonded parameters uniformly to decrease the inaccuracy caused by the direct mixing of UA and CG models that have vastly different Hamiltonians.

Since our mixed representation is designed to model the functional motions of a part of a protein or a protein in a complex with others, it must maintain the stability of various types of protein conformations in a CG protein environment. Although the PACE UA model has been proven to maintain various protein structures reasonably well in a CG water solvent,^48^ whether it can do so in a CG protein environment depends on the quality of its description of interactions across resolutions. Hence, we use the structural stability of proteins or protein complexes as a proxy for indicating the performance of the nonbonded interactions between UA and CG protein sites. The structural stability of proteins has also been used by others to validate nonbonded parameters for CG models.^50,52^ Here, using over 30 experimentally determined protein and complex structures as references for validation, we show that the direct mix of nonbonded parameters with rescaling is suited for coupling the PACE UA and MARTINI CG representations of proteins. We further explore the value ranges of the scaling factors suitable for the parameter adjustment and determine the optimal values for these factors that work for most of the protein systems examined here.

As a demonstration of its applicability for large protein systems, we apply our model to study functional dynamics of Piezo1 protein of the Piezo family.^2,3^ Piezo1 has a central pore domain and three long arms with a spread of hundreds of angstroms. It plays an essential role in converting mechanical stimuli into electrochemical signals by coupling large-scale motions of its arms caused by membrane tension with small-scale motions of pore opening. Only the structure of the closed state of Piezo1 has been resolved.^17^ Due to its large size, only very recently has the dynamics of Piezo1 been studied through AA simulations,^53,54^ in some cases with the help of specialized MD machine Anton.^53^ No CG model can be applied to simulate this system due to its multiscale nature. Our model, on the other hand, can reveal how the central pore is opened when the Piezo1 arms undergo large-scale movement in response to membrane tension, reproducing the observation in the AA simulations with a much lower computational lost. Therefore, our mixed model has a great potential for the study of large complex protein machines with high accuracy and computational efficiency.

## 2 Models and Methods

### 2.1 PACEm Model

In PACEm, there are two levels of representations of systems, one at a UA level and the other adopted from the MARTINI CG model, mapping roughly every four heavy atoms to a CG interaction site. The UA model is only used to represent protein molecules, while the CG model is used to model everything including solvent, lipid, and also protein molecules. How a given system is partitioned into these two types of representation is set at the start of a simulation according to the question to be addressed and remains unchanged throughout the simulation. The overall potential energy of our model is expressed as
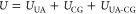
1where *U*_UA_ and *U*_CG_ represent potential energies of UA and CG interactions, respectively. *U*_UA_ has the same functional forms and force field parameters as the original PACE force field,^41^ namely
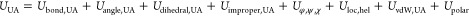
2where the first six terms describe bonded interactions between UA particles, while the remaining terms describe nonbonded interactions between UA particles. *U*_CG_ is the same as that derived for the ELNEDYN22^50^ CG model, a special version of MARTINI22 developed to prevent a large deviation of proteins from their native structures during simulations.^12^ It is expressed as
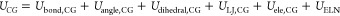
3where there are also the bonded (*U*_bond,CG_, *U*_angle,CG_, and *U*_dihedral,CG_) and the nonbonded terms (*U*_LJ,CG_ and *U*_ele,CG_). The term *U*_ELN_ is used to apply restraints on residual pairs that form native contacts. This elastic network model retains tertiary structures of CG proteins.^50^ The details of the terms in eqs 2 and 3 and their parametrization can be found in refs (12, 44). *U*_UA-CG_ in eq 1 accounts for interactions across the resolutions, and its design and parametrization are the focus of our current study. It also includes the bonded and nonbonded terms. The former term only appears in potential energy functions of intramolecular interactions cut across by interfaces between different levels of representations, while the latter term matters for both intra- and intermolecular interactions.

### 2.2 Coupling between UA and CG Protein Sties

**Figure 1:**
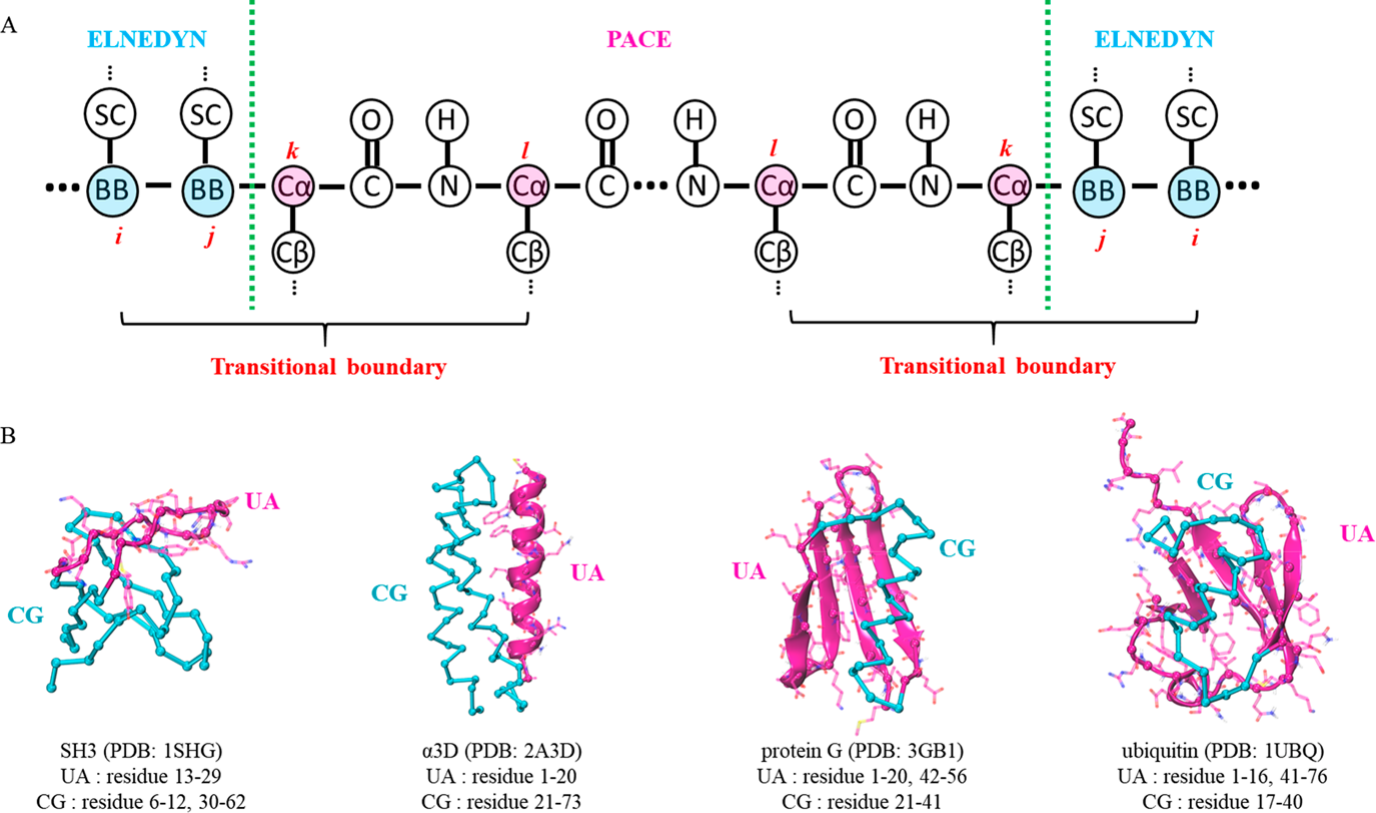
PACEm coupling scheme. (A) Schematic representation of the UA and CG partitioning. (B) PACEm representation of four soluble proteins (magenta: UA region; cyan: CG region).

Inspired by the approach of Kar et al.,^40^ we include different levels of representations in the same molecule by introducing interfaces across resolutions. If the CG part is on the N-terminal side of a residue, the interface must cut across the N–Cα bond of that residue, and all the atoms on the C-terminal side are retained. Similarly, an interface cutting across the Cα–C bond is introduced when the CG part is on the C-terminal side. Since in ELNEDYN the backbone of each residue is represented by a single bead called CG backbone beads (BB) placed at the Cα position of the residue and PACE retained all backbone atoms,^50^ we adopted a straightforward implementation of bonded interactions across the interface between parts with different resolutions. As illustrated in Figure 1A, for each interface, an additional potential energy term *U*_bonded,UA-CG_ was employed to describe interactions involving the sites nearest the interface, including two BB sites (*i*, *j*) on the CG side and two Cα sites (*k*, *l*) on the UA side. *U*_bonded,UA-CG_ is expressed as follows
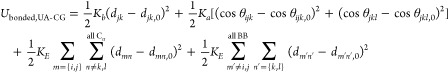
4where *d*_*xy*_ denotes the distance between sites *x* and *y*, θ_*xyz*_ denotes the angle of sites *x*, *y*, and *z*, and *d*_0_ and θ_0_ denote the corresponding distance and angle values, respectively, in the reference structure. Force constants for bond constraints and angular constraints are set to the same values (*K*_*b*_ = 150000 kJ·nm^–2^ mol^–1^ and *K*_*a*_ = 40.0 kJ·mol^–1^) as the ELNEDYN model. To further ensure the structural stability near the interface, the elastic network interactions are applied to any native-contact pair (with 0.5 nm < *d* < 0.9 nm) between the two BB sites (*i* and *j*) and any Cα sites or between any BB sites and the two Cα sites (*k* and *l*). These interactions are defined by the last two terms in eq 4 where the force constant *K*_E_ for an elastic bond is set to the original value (*K*_*E*_ = 500 kJ·nm^–2^·mol^–1^) in ELNEDYN.^50^

The nonbonded interaction energy between a UA particle and a CG particle that are separated by more than two covalent bonds is described with a combination of Lennard-Jones (LJ) and Coulomb potential energy as follows
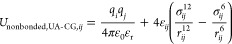
5where ε_*ij*_ denotes the interaction strength of the LJ potential, σ_*ij*_ denotes the distance between two interacting groups with zero interaction energy, *q*_*i*_ and *q*_*j*_ are the partial charges of the two particles taken from the PACE model and the MARTINI model, respectively, and ε_r_ = 15 is an effective dielectric constant that is the same in both the PACE model and the MARTINI model.^12,44^ All the parameters associated with ε_*ij*_ and σ_*ij*_ in eq 5 for protein sites were still missing and thus determined in two steps. First, the initial parameters were generated using the Lorentz–Berthelot combining rule:^55^
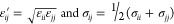
6The respective LJ parameters for sites *i* (ε_*ii*_,σ_*ii*_) and *j* (ε_*jj*_,σ_*jj*_) were taken from PACE and MARTINI. Then, a universal factor γ was applied to tune ε_*ij*_ according to ε_*ij*_ = *γε*_*ij*_^′^.

### 2.3 Coupling between UA Protein Sites and CG Lipids

Apart from the interactions between the UA and CG protein sites, the interactions between the UA protein sites and the CG sites for lipids are also parametrized to enable multiscale simulations in a complex environment like a cell membrane. These interactions are also described in eq 5 where ε_*ij*_ and σ_*ij*_ are to be determined. The original contact distance parameters σ_*ij*_,_old_^49^ were derived with the combining rule, and the original interaction strength parameters ε_*ij*_,_old_ were derived by fitting the model to reproduce partition free energies of small compounds between bulk octanol and water phases and the PMFs of these compounds across model membrane. Whether these parameters need to be tuned further to improve its description of the structures of membrane proteins has not been checked systematically. As such, we used a scaling factor η to tune the new strength parameters ε_*ij*_ = *ηε*_*ij*,old_ for all types of nonbonded interactions between the UA protein sites and the CG lipid sites.

In addition, Jia et al.^56^ showed that the original PACE may underestimate hydrogen bond strength needed to stabilize helical structures in a nonpolar environment like organic solvent and membrane that are known to promote helix formation. To remedy this, we modified the parameters associated with a local HB potential term *U*_loc,hel_ in eq 2. This term is expressed as , where ε_hel_ and σ_hel_ describe a LJ-like attraction applied between the carbonyl oxygen atom in the backbone of residue *i* to the backbone nitrogen atom of residues *i*+3 and *i*+4, intended for strengthening a specific helical turn involving residues *i* to *i*+4. These parameters were optimized to reproduce a proper helical propensity of proteins in solution. Here, we introduced another scaling factor ζ so that helical turns in solution and membrane are stabilized to distinct extents. The new local HB potential term is expressed as
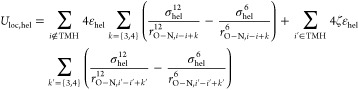
7where *i*′ denotes residues belonging to helices in transmembrane proteins, and *i* denotes helical residues of proteins in solution.

### 2.4 UA/CG Partitioning Scheme of Protein Models

Various protein systems were investigated with our mixed-resolution model. Below are the rules by which each system was divided into UA and CG representations. In general, we avoided introducing the interfaces for switching UA/CG representations within a single helix or a β-sheet comprised of multiple β-strands. Instead, the interfaces were kept by at least two residues away from any residue in these secondary structural elements. Also, we kept the interfaces for resolution switching from cutting across any proline residue.

For the protein systems used for testing and validation, the partitioning of the UA/CG representations was merely aimed for the construction of diverse mixed representations of proteins. For each monomeric protein in the absence of β-sheet, an interface was introduced as close to the middle position in sequence as possible without cutting across any helix. For each monomeric protein with β-sheets, the largest β-sheet structure was represented at one resolution, and the remaining parts of the protein were represented with the other resolution. We selected in total 22 soluble monomeric proteins of varying sizes and folding topologies as benchmarks to test the feasibility of our mixed resolution models for simulations of protein structures. Most of these proteins have been used to examine the ability of other models to maintain the stability of protein structures.^14,40,52^ Of note, for each monomeric protein, we represented the protein in two different ways by swapping the resolutions of the different parts. As such, we complied a collection of 44 constructs of the mixed representations of monomeric proteins (Table S1).

Another nine soluble dimeric complexes were employed to further validate our mixed-resolution model (Table S2). For each case, one of the peptide chains was represented at the UA level, while the other chain was represented with the CG model. The peptide of a smaller size in each heterodimeric complex was always represented at the UA level.

In addition, we validated the applicability of our mixed-resolution model for membrane proteins through simulations of Acetabularia Rhodopsin I (ARI, PDB: 5AX0).^57^ ARI is a monomeric protein, and its UA/CG regions were defined as we did for the soluble monomeric proteins. By swapping the representation resolutions of different parts of the protein, we obtained two distinct constructs of mixed representations of ARI for the validation (Table S1).

**Figure 2:**
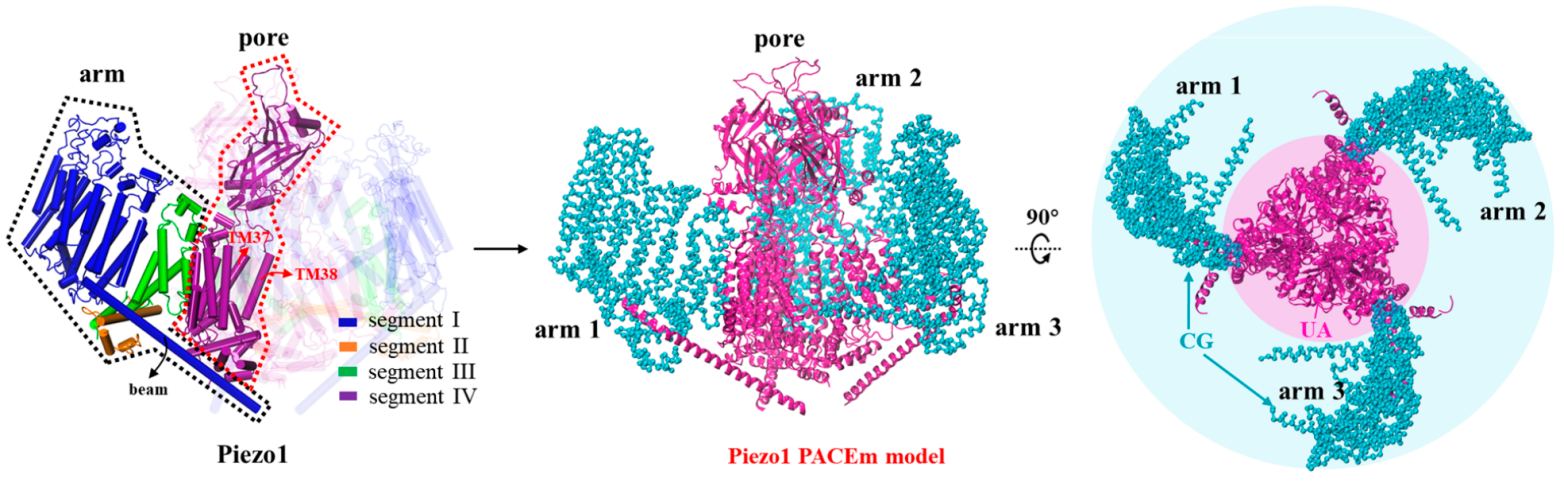
Representation of the Piezo1 PACEm model. The pore region and the three beams (in magenta) were depicted by the PACE UA model. The three arms are depicted by the ELNEDYN CG model (in cyan).

Finally, to study the functional motions of large protein complexes, we determine whether each of protein chains should be represented at either UA, or CG, or the mixed resolution based on the available experimental knowledge on the importance of individual structural elements for the protein function. For instance, our model was employed here to study Piezo1. Piezo1 is a trimeric complex composed of three identical protein chains, each containing over 2500 amino acids. These chains are arranged with a *C*3 symmetry. We constructed our Piezo1 model using the atomistic structure taken from Jiang et al., who have built an atomistic model^53^ based on the cryo-EM structure (PDB: 6B3R)^17^ (Figure 2). Due to the flexibility of Piezo1, only parts of Piezo1 were structurally resolved and included in their atomistic model. Thus, we modeled each Piezo1 chain as four separated segments, namely segment I with residues 782–1365 (Piezo repeat C–F and beam), segment II with residues 1493–1578 (clasp), segment III with residues 1655–1807 (repeat B), and segment IV with residues 1952–2546 (repeat A, anchor, TM37, cap, TM38, and CTD). Therefore, there are 12 separate domains in our Pizeo1 model. The pore region of Pizeo1 is composed of segment IV of each chain. Although these segments are known to be responsible for channel opening, it is unclear what is their subtle structural change that causes the channel opening. As such, these segments were all represented by the UA model. All the other segments are the parts of Pizeo1 arms. They were all represented by the CG model as we are concerned only with the large scale arm motion in response to tension. An exception is segment I harboring a functionally important long helical beam structure^58^ that needs to be represented by the UA model (Figure 2). Thus, the model for segment I had mixed resolutions and was implemented with the intramolecular coupling scheme discussed above.

The example above also shows the importance of available experimental knowledge for a successful application of our model for large protein complexes. This knowledge needs not to be high in resolution as long as it is enough to reveal the functional role of individual domains or structural elements for us to determine UA/CG representations. However, the applicability of our model will be limited in the case of a novel protein where the function of each domain is unknown.

### 2.5 Simulation Settings

All the simulations were performed with the GROMACS v2018 and v2022 software packages.^59^ The summary of the simulation systems investigated in this study is shown in Table S3.

#### AA Simulations

Initial models for AA simulations of protein systems were generated using the CHARMM-GUI Builder.^60^ Each soluble protein was placed in a cubic box filled with TIP3P water,^61^ maintaining a minimum distance of 12 Å from the solute to the box wall. Each membrane protein was embedded into an atomic POPC lipid bilayer. Each system was first neutralized with appropriate quantities of counterions (Na^+^ or Cl^–^) and then salted with 0.15 M NaCl. The CHARMM36m force field^62^ was utilized for proteins, ions, and lipids. Nonbonded interactions were truncated beyond 12 Å. Long-range electrostatic interactions were described with the particle-mesh Ewald method.^63^ An initial 5000-step energy minimization was executed in AA simulations, followed by a 1 ns NPT pre-equilibration simulation with proteins positionally constrained. AA simulations were performed with a 2 fs time step at 310 K using the V-rescale coupling algorithm and 1 bar pressure using isotropic coupling for soluble protein systems or semi-isotropic coupling for membrane protein systems. Each soluble protein system underwent three independent 50 ns AA simulations, and each membrane protein system underwent three independent 100 ns AA simulations.

#### CG Simulations

Four soluble proteins (SH3 domain, α3D, protein G, and ubiquitin) were mapped into the MARTINI22,^12^ ELNEDYN22,^50^ MARTINI3,^11^ and ELNEDYN3^11^ representations using the CHARMM-GUI MARTINI Maker.^64^ Each system was solvated in a MARTINI CG water box, buffered, and neutralized with 150 mM NaCl. LJ forces were truncated at 1.1 nm with the potential shifted to zero at the cutoff using the “potential-modifier” method. Coulomb forces were treated with a reaction field method with a short-range cutoff at 1.1 nm and ϵ_*RF*_ = ∞. In the CG simulations, a 5000-step energy minimization was conducted first, and this was followed by a 1 ns NPT pre-equilibration simulation with the positional constraints on proteins. Finally, all production runs were performed without any positional constraints. The time step was set to 20 fs. The temperature of simulations was set to 323 K using the V-rescale thermostat, and the pressure was set to 1 bar using an isotropic coupling method. Each system underwent three independent 50 ns CG simulations.

#### PACEm Simulations

All proteins in this study were mapped into the PACEm representation using scripts available at https://github.com/hanlab-computChem/hanlab/tree/master/PACEm. A cutoff of 1.2 nm was used for both LJ and Coulombic interactions, with a reaction-field potential applied to the latter. Each system was initially energy minimized for 5000 steps, followed by 1 ns pre-equilibrium simulations with the positional constraints on proteins being gradually relaxed. Finally, all production runs were performed without any positional constraints. The temperature was maintained at 323 K using Nose-Hoover method. The pressure for the soluble proteins was maintained at 1 bar with isotropic conditions and compressibility of 4.5 × 10^–5^ bar^–1^ using the Parrienello-Rahman barostat. The pressure for the membrane proteins was maintained at 1 bar with semi-isotropic conditions and compressibility of 3 × 10^–4^ bar^–1^ using the Parrienello-Rahman barostat. The hydrogen mass repartitioning techniques were employed to increase the mass of all hydrogen sites by four times, allowing for a longer time step of 4 fs in PACEm simulations.^65^

The umbrella sampling method^66^ and the weighted histogram analysis method (WHAM)^67^ were used to calculate the dimerization free energy for dimer complexes. The reaction coordinate was the center of mass (COM) distance between two monomers with a spring constant of 1000 kJ·nm^–2^·mol^–1^. Each system contained at least 13 windows, with the reaction coordinate of each window separated by either 0.1 or 0.2 nm. Each window was simulated for 100 ns.

For the Piezo1 system, a CG model of Piezo1 was initially inserted into a MARTINI POPC bilayer, with dimensions of 300 Å × 300 Å, using the CHARMM-GUI MARTINI Maker.^64^ Following a similar equilibration procedure to previous studies,^53,54^ we first converted the experimental structure of Piezo1 into a ELNEDYN model and then performed the relaxation simulation with harmonic forces restraining the positions of backbone Cα sites of Piezo1. The force constant in this simulation is 1000 kJ·nm^–2^·mol^–1^. This simulation lasted for one microsecond, long enough to fully relax the other part of the system particularly including lipid molecules, allowing for the formation of a downwardly concaved membrane, also known as the inversely dome-shaped structure, caused by Piezo1 in its close state. Next, we converted the equilibrated CG system back into a PACEm system. Specifically, we used the experimental structure to build a PACEm model of Piezo1, aligned its Cα sites with the backbone Cα sites of the equilibrated structure sampled from the restrained CG simulations, and then replaced the protein part of the CG model with the PACEm model while retaining the structural properties of the membrane and solvent from the equilibrated CG system. To investigate the relationship between membrane tension and channel opening, we applied two different pressures to the bilayer XY plane in two separate Piezo1 PACEm systems: +1 bar (representing native pressure) and −10 bar (corresponding to 25.6 mN/m membrane tension). For each pressure condition, we conducted three independent simulations, each lasting 200 ns.

## 3 Results and Discussion

### 3.1 Direct Mixing and Rescaling of UA-CG Interactions Leads to a Balanced Description of Nonbonded Interactions that Maintains Stability of Monomeric Proteins in Mixed Presentations

Given that both PACE and MARTINI include a rich set of site types for describing interactions, a systematic parametrization of nonbonded interactions between the two models would require us to populate a large matrix of interaction parameters. Instead, the parameters can be generated using the Lorentz–Berthelot combining rule (eq 6) to greatly simplify the parametrization process. This technique is a widely applied strategy to enhance the balance among different interaction types. Normally one or a few universal scaling factors are optimized subsequently and used to tune nonbonded interactions to ensure that the model more accurately reflects certain system properties.^68−70^

For soluble proteins, a single scaling factor γ is used to tune all the nonbonded parameters ε_*ij*_. To examine whether this parametrization scheme is feasible and to determine the suitable γ values, we first compiled, as described in Models and Methods, over 40 constructs of mixed representations of soluble monomeric proteins whose structures have been determined. These proteins differ in both sizes and topologies and have served as test cases in both CG and mixed-scale models to evaluate model performance in simulating protein structure and dynamics.^14,40,52^ In each construct, a protein was divided into subunits (see Models and Methods), each of which was represented either by PACE or by MARTINI coupled with the ELNEDYN elastic network potentials. In doing so, we avoided any interface that may disrupt structural integrity of subunits such as a full α-helix, a multistranded β-sheet, or a long loop.

**Figure 3:**
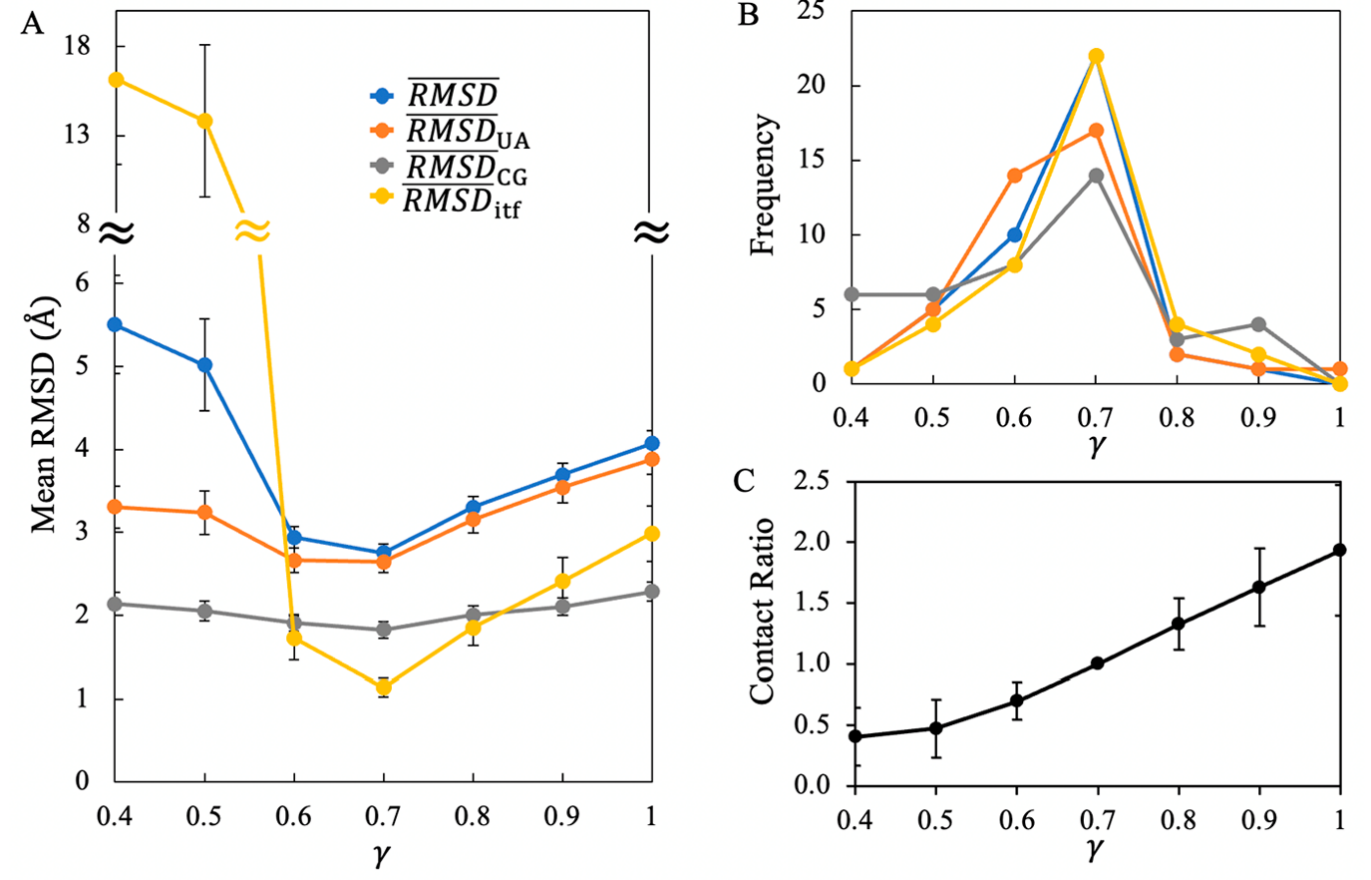
Variation in the structural stability of monomeric proteins in solution with respect to γ. (A) The variations in each type of mean RMSDs with different choices of scaling factor γ. (B) The numbers of constructs that exhibited their least RMSD in simulations at specific γ values. (C) Average counts of backbone contacts between UA and CG subunits over all the constructs examined. A backbone contact occurs for a short distance (less than 6 Å) between a Cα site of a UA subunit and a BB site of a CG subunit. The contacts are normalized with respect to those obtained at γ = 0.7. The error bars indicate the standard errors of the mean.

Our goal here is to test if we can use a single scaling factor to adjust the nonbonded interactions mixed directly from the UA and CG models to ensure that the native structures of the testing proteins remain stable in simulations. Table S4 summarizes the observed root-mean-square deviations (RMSD) from native structures during the simulations of all the constructs. The BB site of each CG residue and the Cα site of each UA residue were used to calculate the RMSD. Since a BB site of the ELNEDYN corresponds to the Cα atom of a residue, our RMSD definitions for the UA and CG parts are consistent with each other. For each construct, the interaction parameters were tuned with different γ values, ranging from 0.4 to 1.0. In each case, the RMSD was found to be sensitive to the value of γ. On average, relatively large RMSD values were observed when γ is either too low () or too high  (Figure 3A). Of the 44 constructs investigated here, there are 41 constructs that could remain stable during at least one of the simulations, exhibiting an RMSD not more than 3.5 Å (Table S4). The average least RMSD for these constructs is 2.6 ± 0.1 Å. Hence, it is indeed feasible to couple UA and CG protein sites within the same proteins by direct mixing and subsequent rescaling of nonbonded parameters.

Intriguingly, for 22 of the 41 constructs, the optimal value of γ was found to be around 0.7 at which the native structures could be well maintained (Figure 3B) with the least RMSD during the simulations with PACEm. In another 10 constructs, the least RMSD was observed at γ = 0.6. Thus, there appears to be an optimal range (*γ ∈* [0.6, 0.7]) where the mixed representation can retain monomeric protein structures. In particular, if γ is fixed at 0.7, it is still possible to obtain an average least RMSD of 2.7 ± 0.1 Å for all the constructs (Figure 3A).

Next, we sought to understand why the variation in γ has such a nonmonotonic effect on the stability of protein structures. For a protein represented with our mixed-resolution model, its stability is affected by the stability of individual subunits at either low or high resolution and that of interfaces across resolutions. To dissect the contribution of different parts to the stability of proteins, we analyzed separately the structural deviation of individual parts of each protein, represented either by the UA (*RMSD*_*UA*_) or CG (*RMSD*_*CG*_) models. The structural deviation (*RMSD*_*itf*_) at the cross-resolution interface was also estimated according to
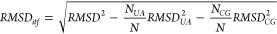
8where *N*_*UA*_ and *N*_*CG*_ denote the number of residues of a protein represented by the UA and CG models, respectively, with *N* = *N*_*UA*_ + *N*_*CG*_.

In the subunits with the CG representation, their secondary and tertiary structures are maintained with the ELNEDYN elastic networks that applied strong harmonic forces to keep native contacts and restrain the proteins in their native conformations.^50^ Thus, the stability of these subunits should be affected little by the change in γ that alters nonbonded interactions between UA and CG protein sites. As expected, *RMSD*_*CG*_ varied within a narrow range (∼1.8–2.3 Å) when γ changed (Figure 3A), and these RMSD values (∼1.5–2.0 Å) are also in accord with those reported previously for several protein systems modeled with the ELNEDYN elastic networks.^50^

On the other hand, the elastic networks are removed at the cross-resolution interfaces. The interfaces are stabilized mainly by the nonbonded interactions formed between UA and CG protein sites, and thus their stability should be vulnerable for the change in γ. As shown in Figure 3A, *RMSD*_*itf*_ indeed varied significantly with γ. Most of the constructs (21 of 41) represented with the mixed model exhibited the lowest RMSD at γ = 0.7 (Figure 3B). Our contact analysis showed that on average more contacts were formed at the interfaces when the nonbonded interactions between UA an CG sites were strengthened with a larger γ (Figure 3C). When γ < 0.7, very large *RMSD*_*itf*_ values were observed, indicating that the UA and CG subunits are detached from each other due to inadequate contacts formed between the two. When γ > 0.7, *RMSD*_*itf*_ also increased due to the formation of additional interfacial contacts that distorted the structures at the interfaces.

As for the subunits represented with the PACE UA model, their structures are stabilized by nonbonded interactions between UA protein sites. Nonetheless, our results showed that the stability of these subunits can still be affected by the change in γ (Figure 3A). On average, the lowest *RMSD*_*UA*_ was calculated to be ∼2.4 Å, and most of these *RMSD*_*UA*_ minima were observed when *γ* ∈ [0.6,0.7] (Figure 3B). In the low γ regime where there is a significant loss of the contacts between the UA and CG subunits (Figure 3C), *RMSD*_*UA*_ was found to increase to ∼3.3 Å, suggesting that the stability of the UA subunits relies on their interaction with their CG counterparts. When γ is large, the large structural deviation of the UA subunits was observed (with *RMSD*_*UA*_ up to ∼3.9 Å), suggesting that the formation of additional contacts (Figure 3C) can distort the structures not only at the UA/CG interfaces but also within the UA subunits. Taken together, our results suggest that the stability of protein structures with the mixed representation is affected both directly and indirectly by the change in the strength of nonbonded interactions between UA and CG protein sites. A balanced strength of these interactions, which can be achieved via rescaling, is crucial for our model to maintain the stability of protein structures.

### 3.2 Comparison of the Performance of PACEm with Other Models on Describing Structures and Dynamics of Monomeric Solution Proteins

We compared the performance of the optimized PACEm model with other force fields (Table 1) using four proteins from our collection of monomeric proteins. These proteins have been used to benchmark the performance of other CG or mixed-resolution models.^40,52^ As expected, the AA CHARMM36m force fields with the TIP3P water model furnished an accurate description of native structures for the four proteins, resulting in a low average RMSD of ∼1.6 Å. In contrast, the protein structures deviated greatly from the native ones with RMSDs ranging from 3 to 16 Å when both versions 2 and 3 of the MARTINI models were employed for simulations. By using the corresponding ELNEDYN versions of these MARTINI models, the native structures of the proteins became much more stable in simulations, showing average RMSDs of 1.6–2.0 Å. The stability gain arose from the use of elastic network that maintains the native contacts with harmonic restraints. These restraints, however, limit the ability of the models to sample conformational fluctuation.

**Table 1:** Average RMSD (Å) of the Backbone for Four Soluble Proteins with Different Force Fields^a^^,^^b^

System	SH3	α3D	protein G	Ubiquitin
CHARMM36m	1.02 ± 0.17	2.81 ± 0.12	0.78 ± 0.07	1.49 ± 0.03
MARTINI22	9.22 ± 0.56	3.31 ± 0.12	7.52 ± 0.58	10.06 ± 1.37
MARTINI3	15.56 ± 0.85	13.98 ± 0.41	14.19 ± 2.06	15.48 ± 1.17
ELNEDYN22	1.07 ± 0.02	1.39 ± 0.03	1.38 ± 0.02	1.82 ± 0.31
ELNEDYN3	1.22 ± 0.08	1.59 ± 0.01	2.21 ± 0.01	2.52 ± 0.23
PACE	2.66 ± 0.42	3.69 ± 0.10	3.02 ± 0.21	2.82 ± 0.03
Part A	2.01 ± 0.21	2.30 ± 0.25	2.35 ± 0.20	2.90 ± 0.10
Part B	2.01 ± 0.38	3.01 ± 0.13	2.23 ± 0.21	1.56 ± 0.05
Interface	1.68 ± 0.10	2.49 ± 0.09	1.90 ± 0.22	1.15 ± 0.17
PACEm	2.01 ± 0.12	2.60 ± 0.25	1.72 ± 0.12	2.48 ± 0.01
Part A (UA)	2.72 ± 0.06	2.49 ± 0.28	1.62 ± 0.06	2.53 ± 0.04
Part B (CG)	1.58 ± 0.11	1.44 ± 0.12	1.04 ± 0.09	0.88 ± 0.02
Interface	0.28 ± 0.14	1.88 ± 0.39	0.95 ± 0.16	1.23 ± 0.03

a All the BB sites and the Cα sites were used for RMSD calculations.

b In each case, the averages and the standard deviations were obtained over three independent 50 ns simulations, the second halves of which were employed for the analysis.

The original PACE model, owing to its UA representation and careful optimization for protein structures, also allowed us to observe the relatively stable native structures of the four proteins without any native restraints. Of these proteins, α3D was the least stable in our simulations, with an ∼3.7 Å RMSD from its native structure. This protein was also the least stable in the AA simulations with its RMSD (∼2.8 Å) considerably higher than the others. The RMSDs for the other three are 2.7–3.0 Å, which is in line with the reported RMSD values (ranging between 1.9 and 3.6 Å with a median of ∼2.5 Å) for a series of monomeric proteins modeled previously with the PACE model.^48^ Of note, we observed considerable higher RMSDs in the simulations with PACE than those obtained from the AA simulations or the CG simulations employing the elastic networks. In fact, relatively larger RMSDs (2.2–5.0 Å) of proteins were also reported in other similar simulations using combined UA/CG models.^33^ Without a highly accurate and detailed description of interactions between protein and solvent or biased forces retaining native protein structures, it remains nontrivial to keep the RMSD of proteins very low in simulations using UA protein models coupled with CG solvent models.

When represented with our PACEm model using γ = 0.7, the RMSDs of the proteins in simulations were largely reduced by 0.85 Å on average. As shown in Table 1, incorporating the ELNEDYN elastic networks to represent a subunit of a protein not only resulted in a more stable structure of this subunit, but it also improved the stability of the interface across resolutions. Although the RMSDs in the simulations with PACEm were still slightly larger than those obtained with the full elastic network models, the native restraints that need to be applied were greatly reduced by ∼30%–70%.

**Figure 4:**
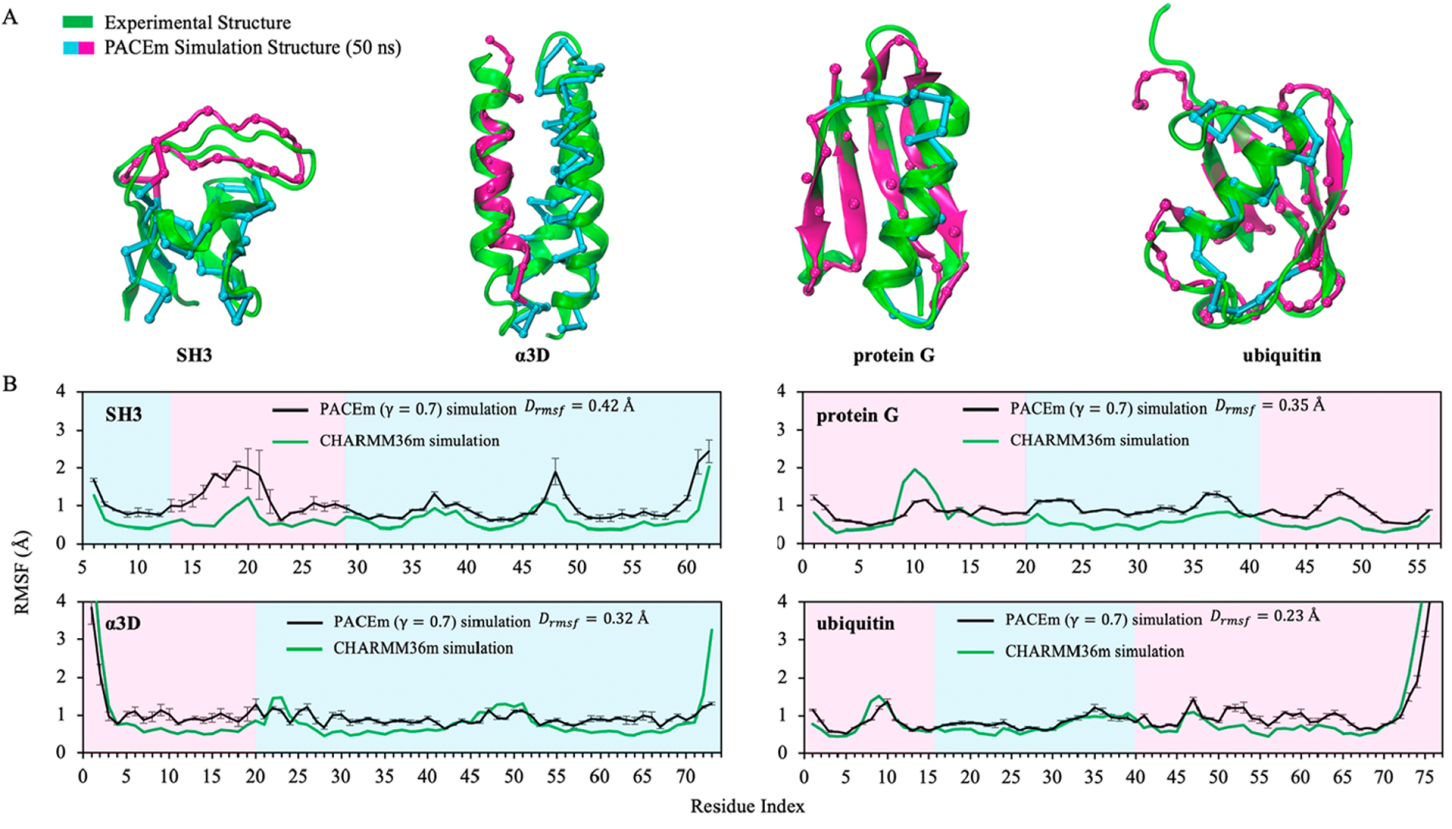
PACEm simulations of four soluble proteins with scaling factor γ **=** 0.7. (A) Protein structure at 50 ns of PACEm simulation with γ = 0.7. The UA and CG regions are colored in magenta and cyan, respectively. The experimental structure is represented in a green cartoon format. (B) RMSF of the backbone (BB/Cα) for four soluble proteins with respect to its experimental structure, plotted against the residue index. Black and green curves denote the RMSF results using PACEm and the CHARMM36m AA force field, respectively. The magenta and cyan areas denote the regions modeled at UA and CG resolutions, respectively. The error bars denote the standard errors of the mean estimated from three independent simulations for each protein.

Figure 4 further shows the root-mean-square fluctuation (RMSF) of different parts of the four proteins modeled with PACEm (black curves). The reference results were obtained with the CHARMM36m/TIP3P AA models, and we calculated the residue-wise difference *D*_*RMSF*,*i*_ = *RMSF*_*i*_^PACEm^ – *RMSF*_*i*_^AA^ between the results from the PACEm and AA simulations. As shown in Figures 4B and S1, for the four proteins, PACEm mostly gave rise to a larger RMSF than the AA model except for several terminal positions of α3D and ubiquitin where the RMSF was smaller, indicating that the proteins in general experienced slightly more fluctuation when modeled with PACEm than with the AA model but the fluctuation of fraying ends of some proteins was underestimated. Of note, although the ELNEDYN elastic networks were applied to the CG parts of the proteins, previous studies showed that this type of elastic networks still allows for the variation in RMSF for different parts of proteins and could be tuned to match a target RMSF.^50^ Also, the RMSF has not been used as a target property to parametrize the PACE UA model. Taken together, our results suggest that there is room for further improvement of both ELNEDYN and PACE, which, however, is beyond the scope of the present study. Despite this discrepancy, the average deviation of RMSF, calculated according to ∑_*i*=1_^*N*^|*D*_*RMSF*,*i*_|/*N*, is 0.23–0.42 Å for the four proteins. Two of the four proteins, namely SH3 and protein G, were also simulated using another mixed-resolution model combining the PRIMO CG model and the CHARMM AA model.^40^ The reported RMSFs for these two proteins deviate from the AA results by 1.96 and 0.75 Å, respectively (Figure S1). Therefore, PACEm still furnishes a reasonable description of the fluctuation of native protein structures.

### 3.3 Structural Stability of Dimeric Proteins in Solution Modeled with the Mixed-Resolution Representation

Next, we examined whether our scheme of resolution mixing is also applicable for dimeric protein complexes in solution. As described in Models and Methods, we created a collection of nine mixed-resolution constructs of homo/heterodimeric protein complexes. In each construct, the smaller proteins were represented at the UA level, while the bigger ones were represented with the CG model. Simulations of these constructs were conducted at different γ values (Table S5). Of these constructs, only the barnase/barstar complex (PDB ID: 1BRS) did not exhibit a RMSD below 4.0 Å for any γ values examined. For the other constructs, the least RMSD was on average 3.4 ± 0.2 Å, 0.8 Å larger than observed for the monomeric solution proteins.

**Figure 5:**
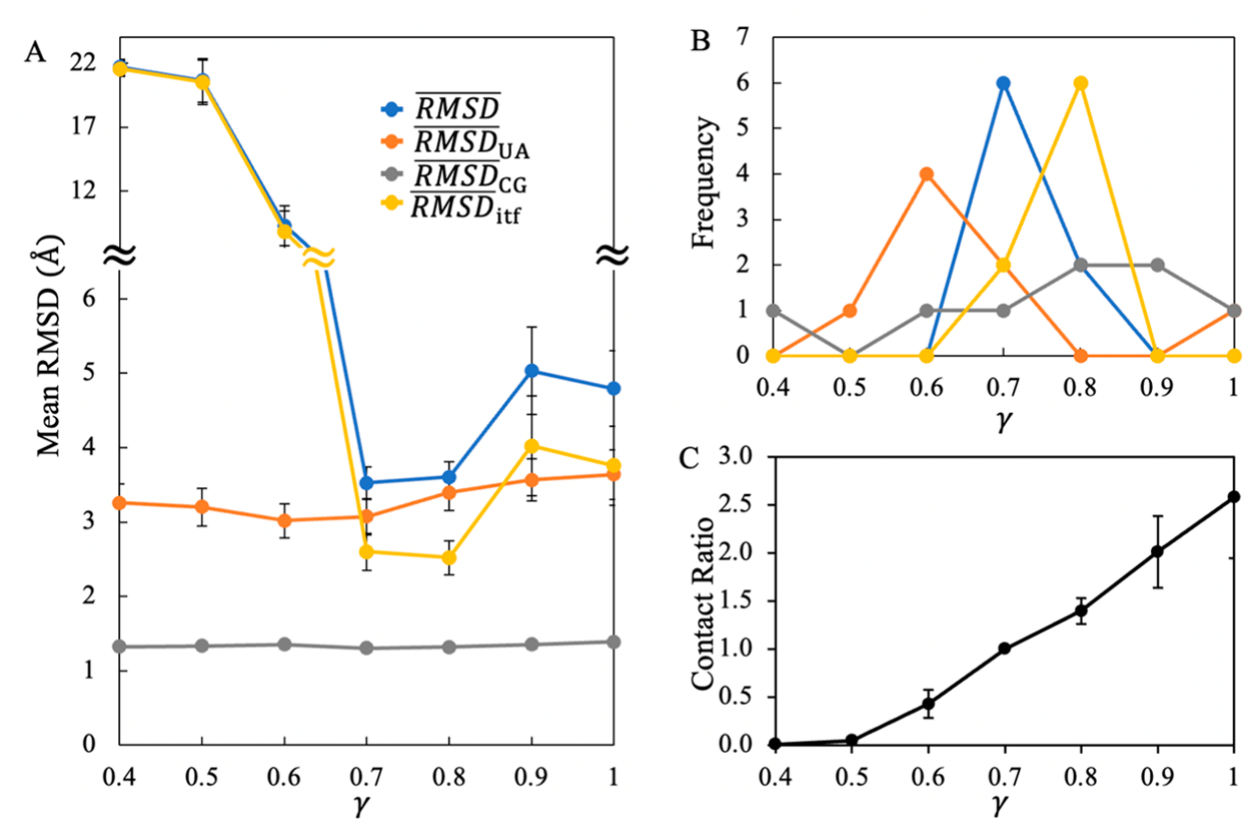
Variation in the structural stability of dimeric protein complexes in solution with respect to γ. (A) The variations in each type of mean RMSDs with different choices of scaling factor γ. (B) The numbers of constructs that exhibited their least RMSD in simulations at specific γ values. (C) Average counts of backbone contacts between UA and CG subunits over all the constructs examined. A backbone contact occurs for a short distance (less than 6 Å) between a Cα site of a UA subunit and a BB site of a CG subunit. The contacts are normalized with respect to those obtained at γ = 0.7. The error bars indicate the standard errors of the mean.

Although the smallest overall RMSDs for the dimeric constructs were the most frequently observed at γ = 0.7 (Figures 5A and 5B), the minimum RMSDs for the UA subunits and for the UA/CG interfaces were mostly observed at γ = 0.6 and γ = 0.8, respectively. It appears that a stronger nonbonded interaction between UA and CG protein sites is needed to maintain the UA/CG interfaces in dimeric complexes than in monomeric proteins, probably due to the lack of any covalent linkages between the UA and CG subunits. As such, when γ decreased from 0.7 to 0.6, the dimeric complexes lost almost half of their interfacial contacts (Figure 5C),and *RMSD*_*itf*_ surged to over 10 Å (Figure 5A); while in the monomeric proteins, the interfaces lost only a quarter of their contacts (Figure 3C), and *RMSD*_*itf*_ remained low (Figure 3A). For most cases, γ = 0.7 is a feasible choice of interaction strength that balances the stability of the UA subunits and UA/CG interfaces in the dimeric constructs, but such a balance may not be always possible for certain dimeric complexes like barnase/barstar (Table S5).

### 3.4 Structure-Based Scheme Also Improves Parameters for Interactions between UA Proteins and CG Lipids

We sought to extend our mixed-resolution model to membrane protein systems. In simulations of such systems, proteins or complexes represented with mixed resolutions should retain their native structures in a CG membrane environment. As a prerequisite, the UA model is required to correctly describe the native protein structures in a CG membrane. As discussed in Models and Methods, we first need to improve the UA model’s ability to maintain the stability of transmembrane helices. Here, we tuned a single parameter ζ that affects the stability of transmembrane helices. As described in eq 7, each helical turn in transmembrane helices can be stabilized by *ζε*_*hel*_ with ε_*hel*_ being used in PACE for the stability gain for a helical turn in solution. We employed three proteins as the model systems, including ARI (PDB: 5AX0),^57^ DAP12 transmembrane domain (PDB: 4WOL),^71^ and potassium channel KcsA (PDB: 1K4C).^72^ They exhibit monomeric, trimeric, and tetrameric structures, respectively, and belong to classic types of membrane proteins, such as G-protein-coupled receptors^57^ and ion channels.^72^ In addition, the stability of transmembrane proteins can also be affected by nonbonded interactions between proteins and lipids. If these interactions were too strong, transmembrane proteins may prefer excessive contacts with surrounding lipid molecules over their own internal native contacts. We conducted simulations to examine the stability of native structures of the three proteins with the strength of interactions between UA proteins and CG lipids being scaled by the factor η (see Models and Methods).

**Figure 6:**
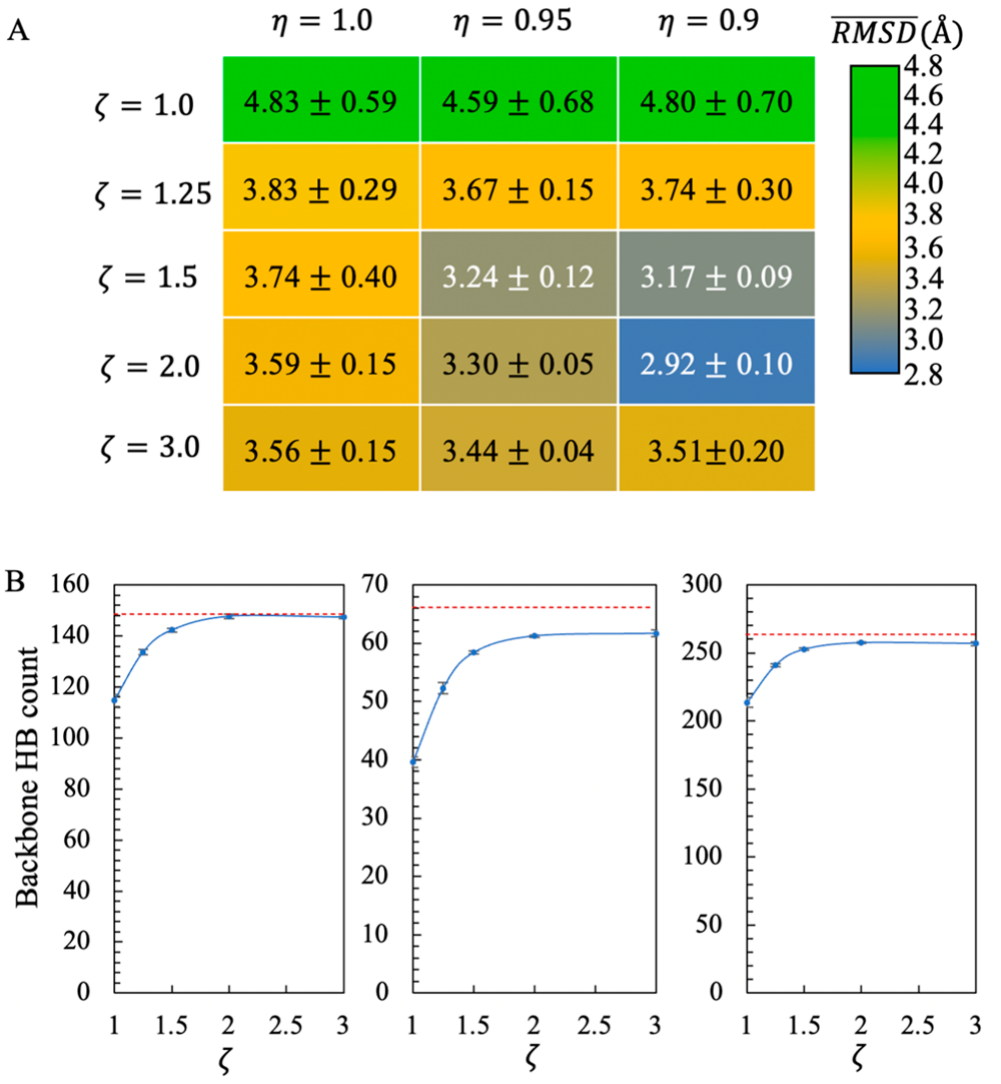
Tuning η and ζ to improve PACEm. (A) The mean RMSD (Å) obtained from simulations of proteins at the UA level in the CG membrane with different (η,ζ) values being used. The mean was averaged over the RMSD values for ARI, DAP12, and KcsA, each of which was averaged over the last halves of three independent simulations. The standard errors of the mean were also provided. (B) Backbone HB counts for ARI (left), DAP12 (middle), and KcsA (right) at different ζ’s. η was set to 0.95. Red dashed lines denote the reference values derived from the CHARMM36m AA simulations.

We tested a set of combinations of η and ζ values with *η* ∈ {0.9,0.95,10} and *ζ* ∈ {1.0,1.25,1.5,2.0,3.0}. The performance of the model with each (η, ζ) pair was assessed according to the average RMSD of the three proteins obtained from simulations (Table S6). Figure 6A plots these RMSD values as a function of η and ζ. The results revealed a low RMSD region for η = 0.95 or 0.9 and ζ = 1.5 or 2.0.

Of note, a value of η other than 1.0 will alter the strength of nonbonded interactions between CG lipid and UA protein sites. The strength of these interactions was originally adjusted to reproduce the potentials of mean force (PMF) of transferring amino acid side chain analogues from solution to a 1,2-dioleoyl-*sn*-glycero-3-phosphocholine (DOPC) membrane.^49^Figure S2A shows the PMFs (*G*(*d*)) as the function of the position of the side chain analogues that were derived using the original nonbonded interaction strength (η = 1.0) and the reduced strength (η = 0.9 or 0.95). These PMFs were compared with those obtained previously using the OPLS/AA model in combination with the Berger lipid force field.^73^ From each PMF, we calculated the free energies of transferring a side chain analogue *x* from solution to either the membrane center (Δ*G*_cen,*x*_) or the position of a local minimum near *d* = 0 (Δ*G*_itf,*x*_), corresponding to the adsorption of the analogue on membrane-water interface. With our original PACE model, we predicted Δ*G*_cen,*x*_ and Δ*G*_itf,*x*_ that differ from the AA results by 1.8 and 1.1 kJ/mol, respectively (Figure S2B). The deviation from the AA results increased moderately to 2.4 kJ/mol for Δ*G*_cen,*x*_ and 2.0 kJ for Δ*G*_itf,*x*_ when η = 0.95, but the deviation increased significantly to 3.4 and 5.3 kJ/mol when η = 0.9. Thus, we did not consider η = 0.9 as a viable parameter choice.

We further examined if the model’s description of transmembrane helices could be improved by parameter tuning. We employed CHARMM36m simulations to calculate the average numbers *N̅*_*hHB*,0_ of helical HBs formed in the transmembrane helical regions of each protein (Figure 6B, green dashed lines). These values were used as the “true” values to compare with the results with our model. We tested the performance of the model for η = 0.95 and ζ varying between 1.0 and 3.0. When ζ = 1.0, *N̅*_*hHB*_ (ζ = 1.0) was significantly lower than *N̅*_*hHB*,0_ for all three proteins. As ζ increased, *N̅*_*hHB*_ (ζ) approached *N̅*_*hHB*,0_ rapidly and appeared to level off until ζ = 1.5. When ζ increased to 2.0, there was only a slight increase in *N̅*_*hHB*_. However, the average RMSD of the three proteins also increased slightly. These results suggest that the parameter sets (η = 0.95, ζ = 1.5) and (η = 0.95, ζ = 2.0) are both feasible for membrane simulations with PACEm, but we chose the set (η = 0.95, ζ = 1.5) as it deviated less from the original PACE model. It should be noted that with both parameter sets, the average RMSD of the three proteins is 3.1–3.2 Å, larger than the RMSD (∼2.5 Å) obtained from the simulations of the same proteins with the CHARMM36m AA model (Table S6). In particular, the RMSDs of ARI and KcsA under PACEm (namely ∼3.0 Å and ∼2.9 Å, respectively) are larger than those under the AA model (1.8 and 2.1 Å, respectively). In addition, for DAP12 and KcsA, *N̅*_*hHB*_ could not reach *N̅*_*hHB*,0_ even when ζ = 3.0. Hence, there is a limit on how much improvement can be achieved for PACEm by tuning η and ζ alone.

**Figure 7:**
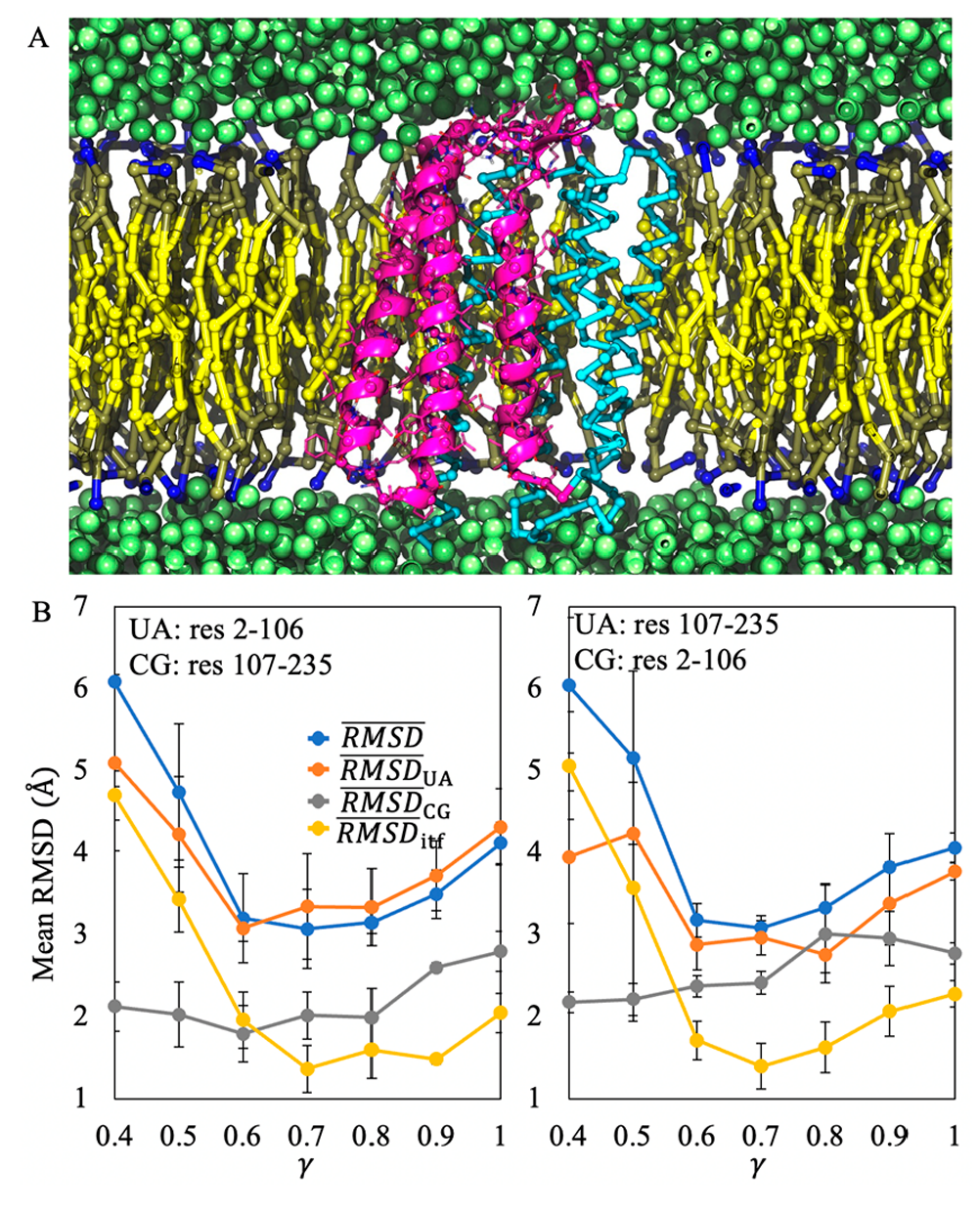
Modeling of ARI with a mixed-resolution representation in the CG membrane. (A) Snapshot of one of the ARI constructs. The UA part of ARI is shown as purple ribbons, and the CG part is shown as cyan sticks. Lipid molecules are shown as yellow sticks, and CG water solvents are shown as green spheres. (B) The variations in each type of mean RMSDs with different choices of scaling factor γ. The two panels denote the results of the two constructs of ARI. The error bars denote the standard errors of the mean over three independent simulations.

Thus far, we have focused on the UA representation of entire proteins in a CG membrane environment. Next, we examine if it is feasible to combine a mixed-resolution protein model with the CG membrane model. In theory, we would need to explore the parameter space of (γ,η,ζ), which is computationally too demanding. Instead, we postulated that the (η,ζ) parameters determined as above and the γ parameter identified for proteins in solution could be combined to describe membrane proteins with mixed resolutions. To test this assumption, we created two constructs of mixed representation of ARI (Figure 7A) as we did for the monomeric proteins in solution (see Models and Methods). We simulated these two constructs at different γ values with η = 0.95 and ζ = 1.5. As shown in Figure 7B, the variations in RMSD for both constructs are not only similar to each other but also similar to those observed for the monomeric proteins in solution (Figure 3A) except that *RMSD*_*itf*_ did not increase drastically at small γ values due to the confinement of a membrane environment. For both constructs, the overall RMSD reached its minimum value of about 3.0 Å when γ = 0.7. Thus, γ = 0.7 appears to be a viable parameter choice for PACEm to model proteins in solutions and in membrane.

### 3.5 Modeling Protein–Protein Interactions in Aqueous and Membrane Environments with PACEm

**Figure 8:**
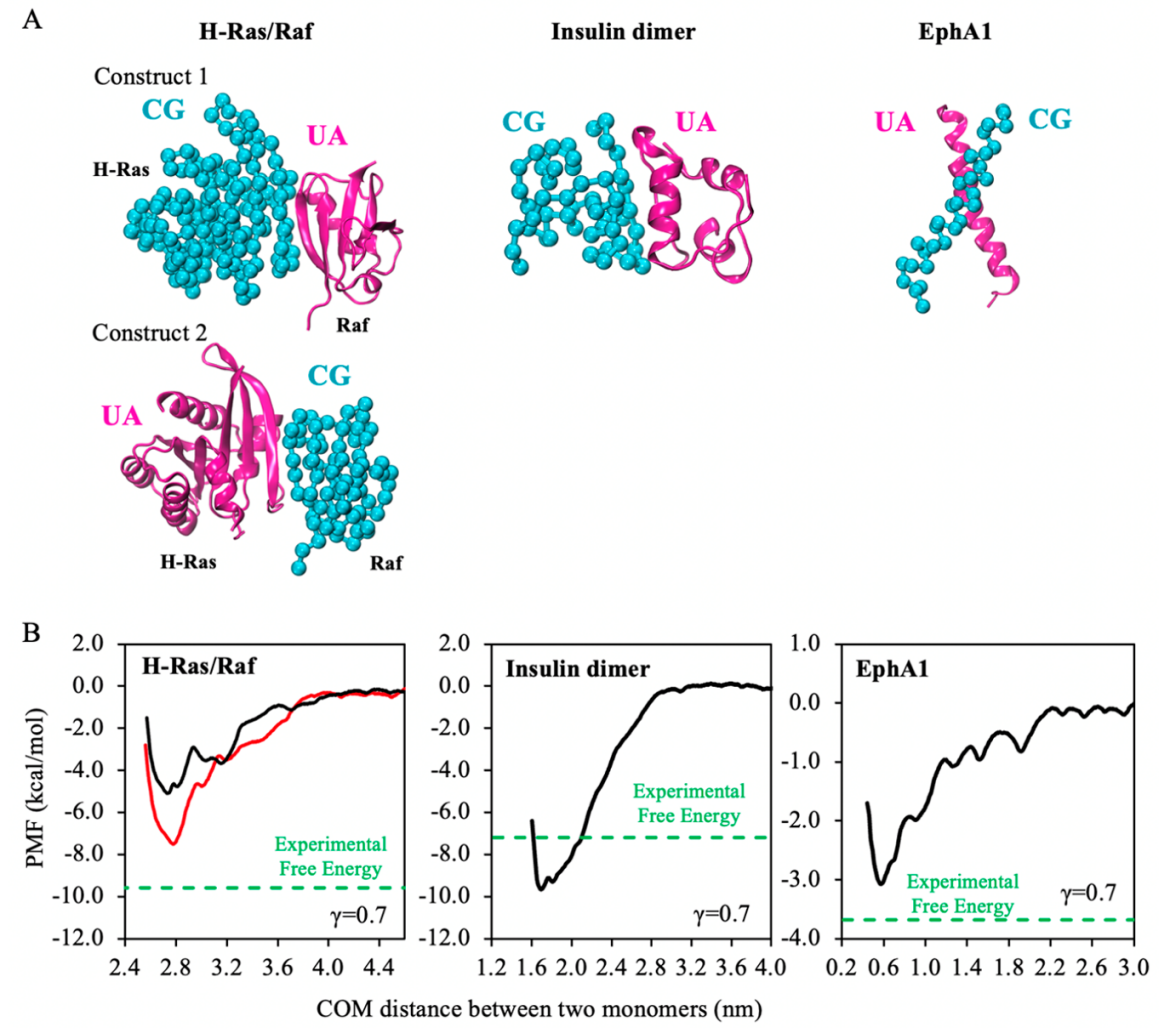
Free energies of association (kcal/mol) for three dimer complexes modeled with PACEm when γ = 0.7. (A) Mixed representation of three protein complexes. The CG monomer is colored cyan, and the UA monomer is colored magenta. (B) The PMFs of complex association. The green dashed line indicates association free energy obtained from experiments. In the results for H-Ras/Raf, the black curve denotes the PMF obtained when the presentation of the complex is construct 1: H-Ras was modeled at the CG resolution, and Raf was modeled at the UA resolution. The red curve denotes the PMF obtained after the representation resolutions of the two domains were swapped (construct 2). For homodimers like insulin dimer and EphA1, only a single PMF can be obtained, which is shown as a black curve.

Protein–protein interactions play a crucial role in many biological processes, including cellular signaling, enzyme regulation, and the formation of protein complexes.^74^ These interactions are primarily governed by nonbonded interactions.^75^ It is thus informative to know how accurate the PACEm can be in describing protein–protein interactions. To this end, we employed PACEm to assess the binding strength of three extensively studied protein complex systems, including H-Ras/Raf,^76^ insulin dimer,^77^ and a transmembrane dimeric complex namely EphA1.^78^ For these dimeric protein complexes, there have been available association free energy data derived experimentally.^79^ For each complex, one interaction partner was represented by the UA model, while the other one was represented by the CG model (Figure 8A). For H-Ras/Raf, there are two possible ways to assign UA/CG representations, both of which were considered. In each case, the umbrella sampling simulation was then conducted to estimate the PMF of association of the two binding partners (see Models and Methods).

For most of dimeric constructs investigated, their structures were the most stable at γ = 0.7 (Figure S3). The PMFs of complex formation were thus calculated at γ = 0.7 (Figure 8B). The depth Δ*G*_A_ of the first free energy well in the PMFs was used as an approximate estimate of binding strength of the complexes (Table 2). For the insulin dimer, the calculated Δ*G*_A_, which is −9.7 kcal/mol, is about 2.5 kcal/mol stronger than experimental value (−7.2 kcal/mol). For H-Ras/Raf, Δ*G*_A_ was calculated to be −5.1 kcal/mol for one construct and −7.5 kcal/mol for the other, both considerably weaker than the experimental value (−9.6 kcal/mol). As for the membrane protein dimer EphA1, Δ*G*_A_ was calculated to be −3.1 kcal/mol, close to the experimental value (−3.7 kcal/mol). These results suggest that the γ value that is the most suited for the description of stable structures of protein complexes does not warrant the reproduction of binding strength of the complexes. To reproduce the binding strength, γ may need to be adjusted separately for individual protein complexes. Although this adjustment is beyond the scope of the present work, our results suggest that γ should be kept in a narrow range (0.6, 0.8): when γ ≤ 0.6, the UA/CG interfaces deformed, as manifested by a large increase in *RMSD*_*itf*_ for all three protein complexes (Figure S3); when γ ≥ 0.8, Δ*G*_A_ was predicted to be much stronger than the experimental values for all the protein complexes (Table 2 and Figure S4).

**Table 2:** Free Energies of Association (kcal/mol) for H-Ras/Raf, Insulin Dimer, and EphA1

	H-Ras/Raf			
Methods	UA subunit: Raf CG subunit: H-Ras	UA subunit: H-Ras CG subunit: Raf	Insulin dimer	EphA1
PACEm simulation (γ = 0.7)	–5.1	–7.5	–9.7	–3.1
PACEm simulation (γ = 0.8)	–18.7	–20.5	–22.0	–14.2
Experiment	–9.6		–7.2	–3.7

### 3.6 Simulations with PACEm Reveal Channel Opening of Piezo1 Induced by Membrane Tension

Given the ability of PACEm to properly describe structures and fluctuations of proteins and interactions between proteins in water and membrane, we further examined its applicability for studying one of super large protein systems, namely Piezo1 (∼7500 amino acids). Piezol has only a relatively small pore domain while most of the remaining parts form three characteristic long curved transmembrane arms, causing the surrounding membrane to form a dome with a spread of over 100 Å.^2,3,18,19^ Notable flattening motions of the arms (by several nanometers) in response to membrane tension or flattening trigger channel pore opening involving movements by several angstroms.^53,54^

We employed PACEm to simulate conformational dynamics of Piezo1. As described in Models and Methods, our representation of Pizeo1 has a central pore region represented with the UA model and three arms represented with the CG model. The initial conformation of the Piezo1 corresponds to a closed state in which the three arms tilted up and caused the formation of an inversed dome. The central pore region sits at the bottom of the inversed dome with its principal axis perpendicular to membrane. The central channel is enclosed by TM37 and TM38 from each of the three protein monomer. These helices packed against each other and occluded the access to the pore. The Piezo1 model was embedded in a 300 × 300 Å^2^ POPC bilayer extending in the XY plane. We started simulations with the closed state and applied two different pressures to the bilayer XY plane of the simulation systems: +1 bar (native pressure) and −10 bar (corresponding to 25.6 mN/m membrane tension). Three independent 200 ns simulations were launched under each pressure condition.

**Figure 9:**
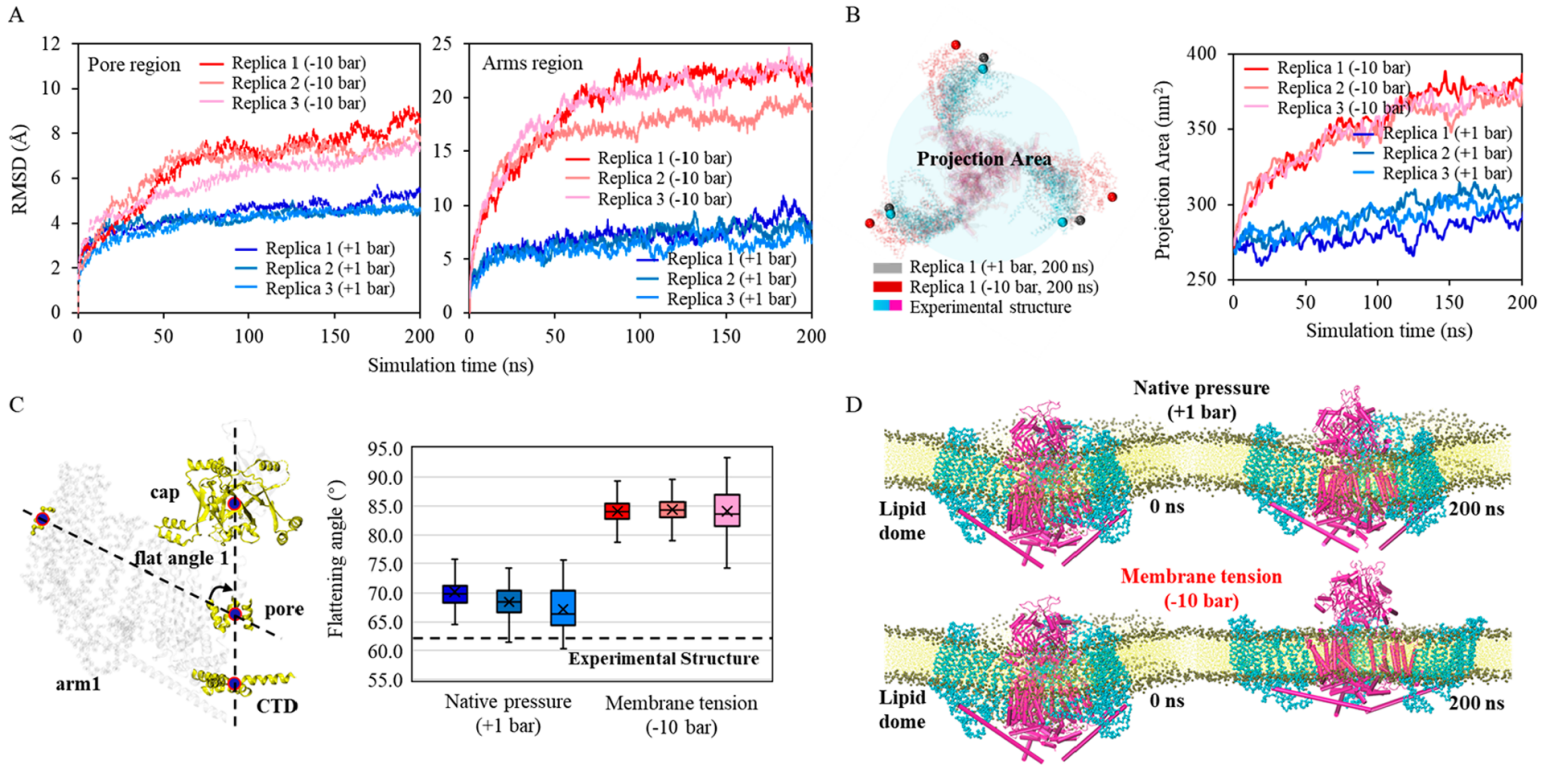
Piezo1 MD simulations with optimized PACEm. (A) Backbone RMSD of the pore and arms regions across three independent native pressure (+1 bar) simulations and three independent membrane tension (−10 bar) simulations. The experimental structures (PDB ID: 6B3R) were used for the RMSD calculations. (B) The projection area of three independent native pressure (+1 bar) simulations and three independent membrane tension (−10 bar) simulations. The area is calculated using the formula π × *r*^2^, where *r* is derived from . Here, *a*, *b*, and *c* represent the distances between the backbone particle from residue ILE859, located within the outermost helix embedded in the bilayer from three Piezo1 arms. The ball marker indicates the position of ILE859. (C) The flattening angle of the three Piezo1 arms across three independent native pressure (+1 bar) simulations and three independent membrane tension (−10 bar) simulations. The angle is defined by the angle between the arm axis (determined by the COM of the outermost helix, residue 850–860, of the arm and the pore region) and the internal axis (determined by the COM of the cap and CTD region), as illustrated on the left. The values of arm 1, arm 2, and arm 3 from the final 100 ns are plotted here. The average value is indicated by the “×” symbol. (D) Snapshots at the 0 and 200 ns of PACEm simulations (replica 1) of Piezo1 under native pressure conditions (top) and membrane tension conditions (bottom), respectively.

Figure 9A shows the time evolution of the RMSD from the cryo-EM structure (PDB ID: 6B3R) in these simulations. At the native pressure (+1 bar), the RMSD values of both the pore and the arms reached a plateau at *t* = ∼50 ns. The stable structures of Piezo1 sampled in the simulations have an RMSD of ∼4.6 Å for the pore and ∼7.5 Å for the arms. Intriguingly, a recent computational study of Piezo1 with AA models also reported the RMSD values of ∼5.0 Å and ∼7.5 Å for the pore and the arms, respectively.^54^ A direct comparison of their RMSD values with ours may not be rigorous as their RMSD values were obtained using a structure subject to an equilibration with the CG model and then calculation with the AA model. Nonetheless, the results shown here suggest that our model can maintain the stable structure of Piezo1, not inferior to the AA model.

We further examined the curvature of the dome by monitoring its projected area.^54^ An increase in the projected area indicates the flattening of the dome. A minor increase (from ∼270 nm^2^ to ∼285 nm^2^) in the projection area was observed at native pressure simulation (curves in cold tone colors, Figure 9B), in line with what should be expected for Piezo1 from the detergent-solubilized conformation to membrane embedded conformation. These data suggest that at native pressure Piezo1 modeled with PACEm can maintain the curved shape of the dome.

When the membrane tension was applied, the projection area expanded to 450 nm^2^, indicating a considerable flattening of the membrane (curves in warm tone colors, Figure 9B). In the meanwhile, the RMSDs for the pore and the arms increased to 7.3 and 20.7 Å, respectively, indicating that both pore and arms responded to the membrane flattening although the induced motion of the arms was much larger than that of the pore. To characterize the large motion of the arms, we measured the tilting angle ω of the arms, defined as the average angle between each arm and the principal axis of the pore (Figure 9C). As shown in Figure 9C, in the simulations at native pressure, the tilting angle was approximately 67°, which is close to the value from the experimental structure (63°). This suggests that the conformation in native pressure simulations retained its curved structure (Figure 9D). However, in the simulations at −10 bar, the tilting angle reached approximately 85°, indicating an almost flat conformation of Piezo1. This finding corroborated the AA simulation results that a flattened membrane forces Piezo1 to adopt a flattened shape (Figure 9D). It is also in line with the experimental finding that an increase in the projection area is a crucial feature for the activation of the Piezo protein family. Therefore, our PACEm can correctly describe the large-scale functional motion of Piezo1 in response to membrane tension.

**Figure 10:**
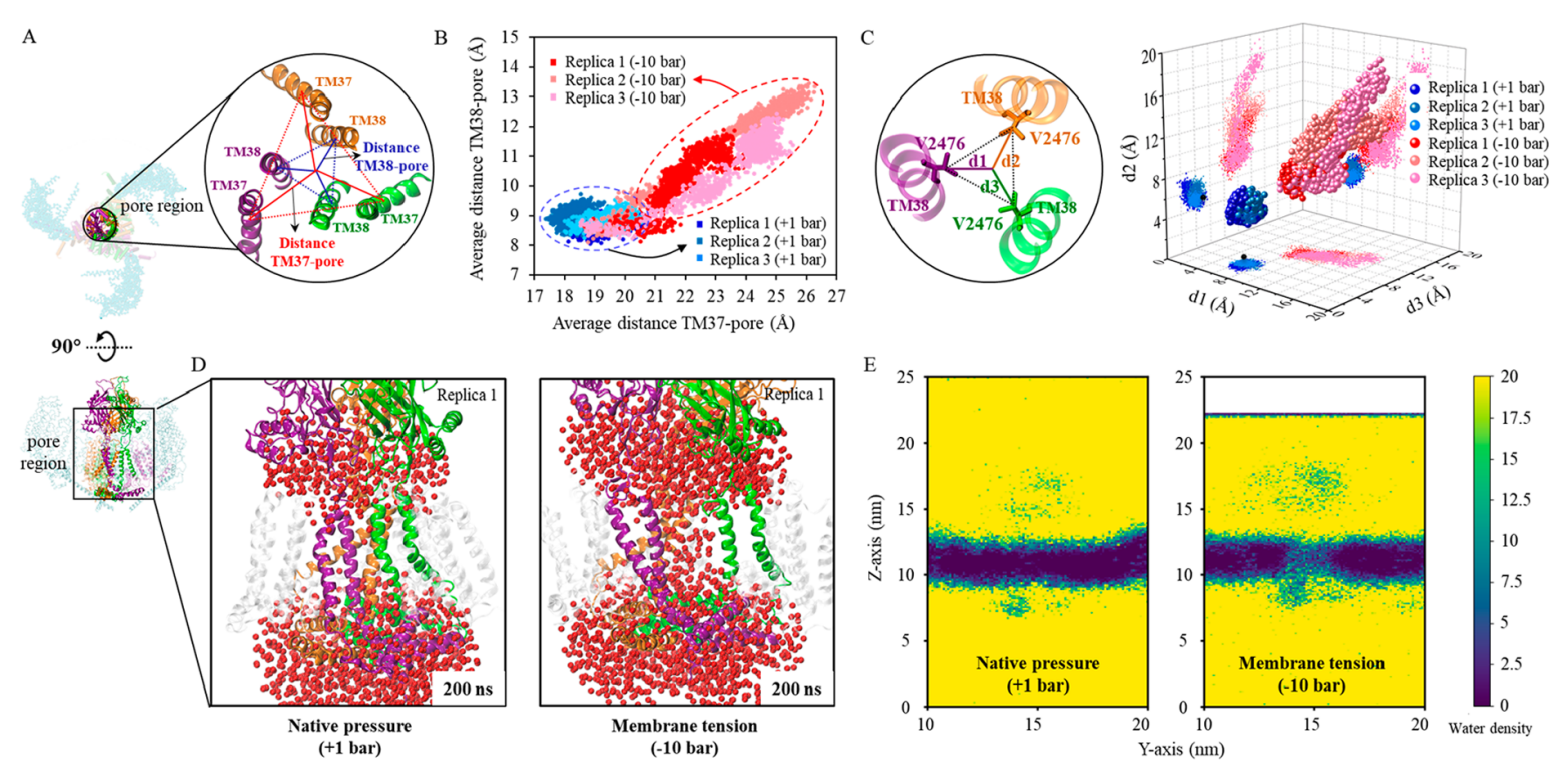
Conformational changes of the Piezo1 pore region. (A) Detailed depiction of the pore region and two types of measures of pore sizes based on the positions of pore-lining helices: either the distances (*d*_1_^37^,*d*_2_^37^,*d*_3_^37^) of the three outer helices TM37 to the pore center or the distances (*d*_1_^38^,*d*_2_^38^,*d*_3_^38^) of the three inner helices TM38 to the pore center. Here, the pore center is defined as the centroid of all TM37s and TM38s, and the centroids of individual TM37s and TM38s are used to calculate their distances to the pore enter. (B) Plots of *d*_37_ = (*d*_1_^37^ + *d*_2_^37^ + *d*_3_^37^)/3 against *d*_38_ = (*d*_1_^38^ + *d*_2_^38^ + *d*_3_^38^)/3 for all the simulations. Each dot corresponds to one snapshot sampled from the simulations, and the dots from different simulations are colored differently. (C) Definition of the pore size based on pore-lining residues V2476: the average distance (*d*_*V*_ = (*d*_1_^V^ + *d*_2_^V^ + *d*_3_^V^)/3) of the Cβ atoms of the three valine residues to the centroid of these Cβ atoms. The panel on the right denotes the plots of (*d*_1_^V^,*d*_2_^V^,*d*_3_^V^) for all the simulations. The black dots denote the distance values calculated based on the experimental structure of the closed state. (D) The water molecules (shown as red spheres) surrounding the pore region under the native pressure (left) and membrane tension (right). The displayed conformations derive from the first independent replica of the two systems at the 200 ns. (E) The number density of water molecules along the Y-Z panel. The density was accumulated using the last 10 frames of three independent trajectories at a 10 ns interval.

Next, we analyzed the conformational change of the pore induced by the membrane tension. Of particular interest is the opening of the pore. Several structural properties have been used to monitor the pore opening, such as the average distance of pore-lining TM37 (*d*_37_) and TM38 (*d*_38_) to the pore center proposed by De Vecchis et al.^54^ and the to-center distance of V2476 (*d*_*V*_) used by Jiang et al.^53^ to determine the size of the bottleneck of the pore (Figure 10A). At the native pressure, all these properties were observed to fluctuate around the values consistent with the experimental structure of the closed states (dots in cold tone colors, Figures 10B and 10C). When the tension was applied, *d*_37_ was observed to increase from <20 Å to 20–30 Å, and *d*_38_ was observed to increase from <10 Å to 10–15 Å (dots in warm tone colors, Figure 10B), consistent with the AA simulation by De Vecchis et al. (cf. Figure 2D in ref (54)). Notably, we observed the Piezo1 pore opening at a lower tension compared to the AA simulation. As a result, membrane integrity was maintained, whereas in the AA simulation, the membrane rupture occurred at 50 ns under −40 bar (cf. Figure 2D in ref (50)). In addition, the hydrophobic gate centroid distance *d*_*v*_ was found to increase above ∼10 Å (dots in warm tone colors, Figure 10C). According to the AA simulation study of Jiang et al.,^53^ a bottleneck with such a diameter can permeate water and ions. Indeed, we did not detect water within the pore region of Piezo1 in our simulations at the native pressure, but CG water was found to fill in and even sometimes pass through the pore in our simulation with membrane tension (Figures 10D and 10E). Therefore, our Piezo1 test case demonstrated that PACEm can accurately capture the coupling between the large motion of the mechanosensitive arm domain and the subtle structural changes associated with the pore opening. Moreover, this gating transition was achieved with a significantly lower computational cost (see below) and under conditions closer to physiological membrane tension.

Of note, although PACEm can describe structures and dynamics of Piezo1 at different levels of details with accuracy almost on a par with its AA counterparts, its computational efficiency is over 35 times higher (Table 3). Thus, this model would be useful especially when extensive sampling of large proteins is needed.

**Table 3:** Comparison of Simulation Efficiency between CHARMM36m, PACEm, and MARTINI^a^

Force field	Box size (nm)	Particle counts	Speed (ns/day)
CHARMM36m	30.0 × 30.0 × 22.4	1906449	10
PACEm^b^	30.0 × 30.0 × 25.0	204243	356
MARTINI^b^	30.0 × 30.0 × 25.0	190425	1811

a Simulation systems contained a Piezo1 embedded in the POPC membrane and solvated in a water box. The simulations were done with a 16-core Intel CPU plus a NVIDIA-RTX4090 graphic card.

b The timesteps for the UA and CG simulations are 4 and 20 fs, respectively. For CG simulations, the entire Piezo1 complex was represented by the MARTINI22 model coupled with the ELNEDYN22 elastic network.

To better demonstrate the advantages of using our mixed resolution model, we used the MARTINI CG model coupled with the ELNEDYN22 elastic network to represent the entire Piezo1 and simulate its dynamics under both native pressure (+1 bar) and membrane tension (−10 bar) conditions. As a much longer time step can be used for the CG model than PACEm (20 fs versus 4 fs), the CG simulations are about four times faster (Table 3). The results shown in Figure S5A reveal that the arm tilting angle of Piezo1 expanded from 70° under native pressure to 80° when tension was applied, which indicates a flatter overall structure. However, the distance at the hydrophobic gate V2476, a critical indicator of the channel’s open or closed state, remained nearly unchanged, with measurements of 4.16 Å under native pressure and 4.91 Å under membrane tension, both indicative of a closed channel (Figure S5B). This observation suggests that while the CG model is effective in portraying general structural changes—like the flattening of Piezo1—it falls short in capturing the finer, yet pivotal, conformational shifts that govern channel opening. Our mixed resolution model is tailored to address this shortcoming by integrating detailed atomistic modeling in critical areas with the simplified CG approach, allowing for a nuanced depiction of both large-scale and subtle molecular events.

## 4 Conclusions

We have developed here a multiscale model called PACEm aimed at tackling challenging tasks of simulations of large protein machines. The model derived combines a UA representation called PACE and the MARTINI coarse-grained (CG) representation in the same simulation systems. A main challenge in developing this model is to determine the parameters for all possible interactions occurring between sites across resolutions. A systematic parametrization of all these interactions is a formidable task. We simplified this task by directly mixing UA/CG parameters and then using universal scaling factors to tune the parameters. Through simulation studies of a collection of protein systems in solution or membrane, we find that this approach is a feasible scheme for developing a mixed-resolution model of proteins and that the stability of native structures of proteins is a sensitive indicator for determining the scaling factors, allowing our model to achieve a balanced description of nonbonded interactions across resolutions. Our model can further be applied in studying functional motion of Piezo1, a complex membrane protein system containing thousands of amino acids with a size of hundreds of angstroms. Our model allows us to capture the subtle motion of the pore opening of Piezo1, a task that only AA models can do but with significantly more computational costs. In summary, our PACEm model demonstrates both accuracy and computational efficiency, establishing it as a promising tool for studying large-scale protein systems.

## Supporting Information Available

**Tables S1** and **S2** present the UA/CG partitioning scheme of protein models in this study. **Table S3** summarizes the simulation systems. **Tables S4** and **S5** show the average RMSD of the backbone (BB/Cα sites) for monomeric proteins and dimeric complexes as determined by PACEm simulations with varying scaling factors γ. **Table S6** shows the average RMSD at Cα sites for three membrane proteins from PACE simulations using different (η, ζ) pairs. **Figure S1** illustrates the average RMSF deviation for four proteins, while **Figure S2** depicts the PMF profiles for amino acid side-chain analogues transitioning from a soluble environment to a DOPC membrane. **Figure S3** displays the variation in mean RMSDs for different scaling factors γ. **Figure S4** presents the PMFs for the association of dimeric complexes, and **Figure S5** shows the simulation results of Piezo1 employing the MARTINI CG model coupled with the ELNEDYN22 elastic network (PDF)
