# Supplementary material for: Simulations of Functional Motions of Super Large Biomolecules with a Mixed-Resolution Model
Source: J Chem Theory Comput. 2024 Feb 20;20(5):2228–45. doi: 10.1021/acs.jctc.3c01046 (PMC10938502; doi:10.1021/acs.jctc.3c01046)
Supplement: Supplementary file 1 — ct3c01046_si_001.pdf [file ct3c01046_si_001.pdf]

Simulations of Functional Motions of Super Large Biomolecules with a Mixed-Resolution Model

Shu Li[a,b,#,*], Bo Hua Wu[b,#], Yun Lyna Luo[e], Wei Han[b,c,d,*]

[a] *Centre for Artificial Intelligence Driven Drug Discovery, Faculty of Applied Sciences, Macao Polytechnic University, Macao 999078, China*

[b] *State Key Laboratory of Chemical Oncogenomics, Guangdong Provincial Key Laboratory of Chemical Genomics, School of Chemical Biology and Biotechnology, Peking University Shenzhen Graduate School, Shenzhen 518055, China*

[c] *Department of Chemistry, Faculty of Science, Hong Kong Baptist University, Hong Kong SAR 999077, China*

[d] *Institute of Chemical Biology, Shenzhen Bay Laboratory, Shenzhen 518132, China*

[e] *Department of Biotechnology and Pharmaceutical Sciences, Western University of Health Sciences, Pomona, California 91766, United States*

[#] *Shu Li and Bo Hua Wu contributed equally to this paper.*

* Email: shuli@mpu.edu.mo, hanw_chem@hkbu.edu.hk

Supporting Information

Table S1. The UA/CG partitioning scheme for monomeric proteins.

PDB	Residue number	Construct	UA region	CG region
1SHG	50	1	13-29	6-12, 30-62
		2	6-12, 30-62	13-29
1GYV	120	1	742-757, 791-822	703-741, 758-790
		2	703-741, 758-790	742-757, 791-822
2E3H	76	1	211-246, 270-300	245-269
		2	245-269	211-246, 270-300
1PGB	61	1	1-10, 28-61	11-27
		2	11-27	1-10, 28-61
2O31	67	1	818-827, 845-884	828-844
		2	828-844	818-827, 845-884
1XX8	66	1	16-50	1-17, 51-66
		2	1-17, 51-66	16-50
1TQG	105	1	0-33	34-104
		2	34-104	0-33
1R69	63	1	1-38	39-63
		2	39-63	1-38
2CKX	83	1	578-603	604-660
		2	604-660	578-603
1BKR	108	1	2-56	57-109
		2	57-109	2-56
2A3D	73	1	1-20	21-73
		2	21-73	1-20
3GB1	56	1	1-20, 42-56	21-41
		2	21-41	1-20, 42-56
1UBQ	76	1	1-16,41-76	17-40
		2	17-40	1-16,41-76
1RA4	117	1	28-33, 46-54, 73-79, 94-103	4-27, 34-45, 55-72, 80-93, 104-120
		2	4-27, 34-45, 55-72, 80-93, 104-120	28-33, 46-54, 73-79, 94-103
2KYR	108	1	1-14, 34-49, 58-67, 78-84	15-33, 50-57, 68-77, 85-108
		2	15-33, 50-57, 68-77, 85-108	1-14, 34-49, 58-67, 78-84
2VIM	104	1	1-5, 19-30, 49-60, 70-91	6-18, 31-48, 61-69, 91-104
		2	6-18, 31-48, 61-69, 91-104	1-5, 19-30, 49-60, 70-91
1CRN	46	1	1-6, 32-46	7-31
		2	7-31	1-6, 32-46
1ORC	64	1	39-61	3-38
		2	3-38	39-61
1PGB	56	1	1-20, 40-56	21-39

Table S1. (continued)

PDB	Residue number	Construct	UA region	CG region
1PGB	56	2	21-39	1-20, 40-56
2UIG	110	1	11-17, 31-71	3-10, 18-30
		2	3-10, 18-30	11-17, 31-71
1QYO	236	1	2-49, 92-237	50-91
		2	50-91	2-49, 92-237
1OPS	64	1	2-26, 42-65	27-41
		2	27-41	2-26, 42-65
5AX0 [a]	234	1	2-106	107-235
		2	107-235	2-106

a) Membrane monomeric protein.

Table S2. The UA/CG partitioning scheme for dimeric complexes.

PDB	Residue number	UA region	CG region
4C4K	191	chain A (94)	chain B (97)
2VLN	214	chain A (80)	chain B (134)
1VET	240	chain A (122)	chain B (118)
4JW3	208	chain C (97)	chain A (111)
5ELU	166	chain B (75)	chain A (91)
6GB2	255	chain A (126)	chain B (129)
4GON	244	chain B (78)	chain A (166)
1BRS	197	chain D (89)	chain A (108)
4INS	102	chain A B (51)	chain C D (51)

Table S3. Summary of simulation systems investigated in the present study.

System	PDB	Model	Box size (nm)	Particles[a]	Simulation time (ns)
monomeric protein	SH3 (1SHG)	CHARMM36m	5.4 × 5.4 × 5.4	14554	3 × 50
		ELNEDYN22	5.6 × 5.6 × 5.6	1488	3 × 50
		MARTINI22	5.6 × 5.6 × 5.6	1492	3 × 50
		ELNEDYN3	5.6 × 5.6 × 5.6	1503	3 × 50
		MARTINI3	5.6 × 5.6 × 5.6	1500	3 × 50
		PACE	5.6 × 5.6 × 5.6	1893	3 × 50
		PACEm	5.6 × 5.6 × 5.6	1605 (1768)	2 × 7 × 3 × 50
	α3D (2A3D)	CHARMM36m	6.4 × 6.4 × 6.4	24666	3 × 50
		ELNEDYN22	6.6 × 6.6 × 6.6	2447	3 × 50
		MARTINI22	6.6 × 6.6 × 6.6	2437	3 × 50
		ELNEDYN3	6.6 × 6.6 × 6.6	2454	3 × 50
		MARTINI3	6.6 × 6.6 × 6.6	2449	3 × 50
		PACE	6.6 × 6.6 × 6.6	2952	3 × 50
		PACEm	6.6 × 6.6 × 6.6	2586 (5009)	2 × 7 × 3 × 50
	protein G (3GB1)	CHARMM36m	5.7 × 5.7 × 5.7	17275	3 × 50
		ELNEDYN22	5.7 × 5.7 × 5.7	1567	3 × 50
		MARTINI22	5.7 × 5.7 × 5.7	1572	3 × 50
		ELNEDYN3	5.7 × 5.7 × 5.7	1574	3 × 50
		MARTINI3	5.7 × 5.7 × 5.7	1578	3 × 50
		PACE	5.7 × 5.7 × 5.7	1946	3 × 50
		PACEm	5.7 × 5.7 × 5.7	1812 (1703)	2 × 7 × 3 × 50
	Ubiquitin (1UBQ)	CHARMM36m	6.8 × 6.8 × 6.8	29414	3 × 50
		ELNEDYN22	6.5 × 6.5 × 6.5	2339	3 × 50
		MARTINI22	6.5 × 6.5 × 6.5	2337	3 × 50
		ELNEDYN3	6.5 × 6.5 × 6.5	2339	3 × 50
		MARTINI3	6.5 × 6.5 × 6.5	2339	3 × 50
		PACE	6.5 × 6.5 × 6.5	2867	3 × 50
		PACEm	6.5 × 6.5 × 6.5	2700 (2498)	2 × 7 × 3 × 50
	1TQG	PACEm	7.4 × 7.4 × 7.4	3719 (3950)	2 × 7 × 3 × 50
	1R69	PACEm	5.5 × 5.5 × 5.5	1699 (1598)	2 × 7 × 3 × 50
	2CKX	PACEm	6.3 × 6.3 × 6.3	2398 (2489)	2 × 7 × 3 × 50
	1BKR	PACEm	6.5 × 6.5 × 6.5	2785 (2778)	2 × 7 × 3 × 50
	1RA4	PACEm	6.3 × 6.3 × 6.3	2362 (2725)	2 × 7 × 3 × 50

Table S3. (continued)

System	PDB	Model	Box size (nm)	Particles	Simulation time (ns)
monomeric protein	2KYR	PACEm	6.7 × 6.7 × 6.7	2883 (2992)	2 × 7 × 3 × 50
	2VIM	PACEm	5.8 × 5.8 × 5.8	2045 (2064)	2 × 7 × 3 × 50
	1CRN	PACEm	5.3 × 5.3 × 5.3	1387 (1426)	2 × 7 × 3 × 50
	1ORC	PACEm	5.5 × 5.5 × 5.5	1618 (1663)	2 × 7 × 3 × 50
	1PGB	PACEm	5.7 × 5.7 × 5.7	1818 (1697)	2 × 7 × 3 × 50
	2IUG	PACEm	6.6 × 6.6 × 6.6	2808 (2844)	2 × 7 × 3 × 50
	1QYO	PACEm	8.4 × 8.4 × 8.4	6478 (5417)	2 × 7 × 3 × 50
	1OPS	PACEm	5.5 × 5.5 × 5.5	1743 (1523)	2 × 7 × 3 × 50
	1GYV	PACEm	7.1 × 7.1 × 7.1	3418 (3569)	2 × 7 × 3 × 50
	2E3H	PACEm	7.0 × 7.0 × 7.0	3359 (3088)	2 × 7 × 3 × 50
	1PWT	PACEm	5.8 × 5.8 × 5.8	1978 (1794)	2 × 7 × 3 × 50
	2O31	PACEm	5.8 × 5.8 × 5.8	2023 (1800)	2 × 7 × 3 × 50
	1XX8	PACEm	5.8 × 5.8 × 5.8	1913 (1908)	2 × 7 × 3 × 50
dimeric complex	4C4K	PACEm	7.8 × 7.8 × 7.8	4773	7 × 3 × 50
	2VLN	PACEm	8.4 × 8.4 × 8.4	5655	7 × 3 × 50
	1VET	PACEm	9.3 × 9.3 × 9.3	7686	7 × 3 × 50
	4JW3	PACEm	7.6 × 7.6 × 7.6	4487	7 × 3 × 50
	5ELU	PACEm	8.0 × 8.0 × 8.0	4910	7 × 3 × 50
	6GB2	PACEm	9.5 × 9.5 × 9.5	8255	7 × 3 × 50
	H-Ras/Raf (4GON)	PACEm	11.1 × 11.1 × 11.1	12105 (12718)	2 × 7 × 3 × 50
		PACEm (umbrella sampling)	11.1 × 11.1 × 11.1	12105 (12718)	4 × 14 × 100
	Barnase/Barstar (1BRS)	PACEm	11.7 × 11.7 × 11.7	14115 (14227)	2 × 7 × 3 × 50
		PACEm (umbrella sampling)	11.7 × 11.7 × 11.7	14115 (14227)	4 × 14 × 100
	Insulin (4INS)	PACEm	11.1 × 11.1 × 11.1	11796	7 × 3 × 50
		PACEm (umbrella sampling)	11.1 × 11.1 × 11.1	11796	2 × 14 × 100
	EphA1 (2K1L)	PACEm	11.5 × 11.5 × 11.3	12540	7 × 3 × 50
		PACEm (umbrella sampling)	11.5 × 11.5 × 11.3	12540	4 × 14 × 100

Table S3. (continued)

<table>
<tr><th>System</th><th>PDB</th><th>Model</th><th>Box size (nm)</th><th>Particles</th><th>Simulation time (ns)</th></tr>
<tr><td rowspan="13">membrane protein systems</td><td rowspan="3">ARI (5AX0)</td><td>CHARMM36m</td><td>8.0 × 8.0 × 10.2</td><td>60473</td><td>3 × 100</td></tr>
<tr><td>PACE</td><td>8.0 × 8.0 × 9.2</td><td>6388</td><td>5 × 3 × 100</td></tr>
<tr><td>PACEm</td><td>8.0 × 8.0 × 9.2</td><td>5531 (5678)</td><td>2 × 7 × 3 × 100</td></tr>
<tr><td rowspan="2">DAP12 (4WOL)</td><td>CHARMM36m</td><td>5.0 × 5.0 × 8.9</td><td>20504</td><td>3 × 100</td></tr>
<tr><td>PACE</td><td>5.0 × 5.0 × 8.7</td><td>2370</td><td>5 × 3 × 100</td></tr>
<tr><td rowspan="2">KcsA (1K4C)</td><td>CHARMM36m</td><td>8.0 × 8.0 × 10.7</td><td>63100</td><td>3 × 100</td></tr>
<tr><td>PACE</td><td>8.0 × 8.0 × 10.6</td><td>8292</td><td>5 × 3 × 100</td></tr>
<tr><td>amino acid sidechain analogues from solute to DOPC</td><td>PACEm (umbrella sampling)</td><td>/</td><td>/</td><td>3 × 17 × 45 × 20</td></tr>
<tr><td rowspan="3">Piezo1</td><td>CHARMM36m</td><td>30.0 × 30.0 × 22.4</td><td>1906449</td><td>/</td></tr>
<tr><td>ELNEDYN22</td><td>30.0 × 30.0 × 25.0</td><td>190425</td><td>6 × 200</td></tr>
<tr><td>PACEm</td><td>30.0 × 30.0 × 25.0</td><td>204243</td><td>6 × 200</td></tr>
</table>

a) Two particle counts are listed for the protein that has two types of PACEm constructs. The unbracketed number represents the particle count in construct 1, while the bracketed one indicates the particle count in construct 2.

Table S4. The average RMSD (Å) of the backbone (BB/Cα sites) for 22 monomeric soluble proteins using PACEm with different choices of scaling factor γ.[a, b, c]

PDB	γ	construct 1				construct 2			
		All	UA subunit	CG subunit	Interface	All	UA subunit	CG subunit	Interface
1SHG	1	4.4 ± 0.1	2.5 ± 0.2	1.6 ± 0.1	4.0 ± 0.1	2.5 ± 0.1	2.2 ± 0.2	1.9 ± 0.1	0.6 ± 0.1
	0.9	3.3 ± 0.3	3.1 ± 0.4	1.7 ± 0.0	2.4 ± 0.3	2.4 ± 0.1	1.9 ± 0.1	2.1 ± 0.1	0.6 ± 0.0
	0.8	2.7 ± 0.5	2.8 ± 0.2	1.5 ± 0.1	1.8 ± 0.3	2.3 ± 0.1	1.7 ± 0.1	2.0 ± 0.1	0.9 ± 0.1
	0.7	2.0 ± 0.1	2.7 ± 0.1	1.6 ± 0.1	0.3 ± 0.1	2.1 ± 0.0	1.6 ± 0.1	2.1 ± 0.1	0.2 ± 0.0
	0.6	2.0 ± 0.1	2.6 ± 0.5	1.7 ± 0.1	0.4 ± 0.3	2.3 ± 0.5	1.5 ± 0.0	1.8 ± 0.3	1.4 ± 0.7
	0.5	3.4 ± 0.1	2.5 ± 0.1	1.4 ± 0.0	2.8 ± 0.1	3.4 ± 0.2	1.5 ± 0.1	2.5 ± 0.1	2.3 ± 0.2
	0.4	3.8 ± 0.1	3.0 ± 0.2	1.5 ± 0.0	3.1 ± 0.2	3.8 ± 0.2	1.6 ± 0.0	2.4 ± 0.2	3.0 ± 0.2
1GYV	1	3.9 ± 0.4	4.0 ± 0.5	3.0 ± 0.3	2.0 ± 0.6	4.7 ± 0.9	4.7 ± 1.0	3.3 ± 0.5	2.7 ± 1.1
	0.9	3.0 ± 0.3	2.9 ± 0.5	2.3 ± 0.2	1.3 ± 0.4	4.5 ± 0.4	4.6 ± 0.6	3.0 ± 0.1	1.9 ± 0.1
	0.8	3.3 ± 0.3	3.3 ± 0.5	2.3 ± 0.3	1.3 ± 0.3	3.6 ± 0.4	3.4 ± 0.4	2.9 ± 0.3	1.1 ± 0.6
	0.7	2.8 ± 0.1	2.9 ± 0.1	2.2 ± 0.2	0.7 ± 0.1	2.5 ± 0.3	2.4 ± 0.3	2.2 ± 0.2	0.5 ± 0.2
	0.6	2.8 ± 0.3	2.7 ± 0.3	2.2 ± 0.2	1.1 ± 0.3	3.1 ± 0.4	2.4 ± 0.5	3.2 ± 0.2	1.1 ± 0.6
	0.5	4.7 ± 0.5	3.7 ± 0.4	3.1 ± 0.4	5.4 ± 1.2	4.8 ± 0.3	3.8 ± 0.2	4.4 ± 0.3	3.3 ± 0.4
	0.4	5.5 ± 0.3	3.8 ± 0.3	3.5 ± 0.1	8.6 ± 1.1	5.3 ± 0.7	3.9 ± 0.4	4.9 ± 0.8	4.7 ± 1.1
2E3H[e]	1	7.0 ± 0.9	7.4 ± 1.0	1.4 ± 0.0	4.1 ± 0.8	4.1 ± 0.5	3.7 ± 0.1	2.3 ± 0.2	4.9 ± 2.0
	0.9	6.7 ± 0.4	6.8 ± 0.4	1.6 ± 0.1	4.7 ± 1.2	3.0 ± 0.1	2.8 ± 0.4	2.3 ± 0.2	1.5 ± 0.2
	0.8	6.3 ± 0.6	6.6 ± 0.7	1.9 ± 0.1	3.5 ± 0.7	2.7 ± 0.3	2.3 ± 0.3	2.0 ± 0.1	1.5 ± 0.6
	0.7	6.5 ± 0.3	6.8 ± 0.3	1.5 ± 0.0	3.4 ± 0.4	3.1 ± 0.1	3.0 ± 0.3	2.1 ± 0.2	1.9 ± 0.5
	0.6	6.6 ± 0.4	7.0 ± 0.4	1.4 ± 0.1	3.5 ± 0.8	3.1 ± 0.1	3.3 ± 0.2	2.0 ± 0.2	1.8 ± 0.1
	0.5	7.0 ± 0.8	7.2 ± 0.8	1.5 ± 0.0	5.1 ± 1.4	3.1 ± 0.2	2.8 ± 0.4	2.2 ± 0.2	2.0 ± 0.3
	0.4	6.5 ± 0.7	6.5 ± 0.7	1.5 ± 0.0	5.2 ± 1.5	3.0 ± 0.3	2.3 ± 0.3	1.9 ± 0.1	2.5 ± 0.6
1PVT	1	3.6 ± 0.2	3.4 ± 0.2	2.2 ± 0.1	1.4 ± 0.2	3.3 ± 0.1	2.4 ± 0.3	2.3 ± 0.2	2.6 ± 0.4
	0.9	3.7 ± 0.1	3.7 ± 0.2	2.0 ± 0.1	1.4 ± 0.2	3.2 ± 0.2	2.7 ± 0.0	1.9 ± 0.1	2.7 ± 0.5
	0.8	3.8 ± 0.4	3.6 ± 0.5	2.0 ± 0.1	2.0 ± 0.3	3.3 ± 0.2	2.6 ± 0.1	2.6 ± 0.3	2.1 ± 0.3
	0.7	3.2 ± 0.3	3.0 ± 0.3	2.0 ± 0.1	1.2 ± 0.2	2.7 ± 0.2	2.2 ± 0.2	2.2 ± 0.3	1.1 ± 0.1
	0.6	3.6 ± 0.4	3.2 ± 0.2	2.1 ± 0.2	2.2 ± 1.1	2.9 ± 0.2	2.3 ± 0.2	2.1 ± 0.1	1.8 ± 0.5
	0.5	4.8 ± 0.4	3.2 ± 0.1	2.6 ± 0.2	6.9 ± 1.6	6.0 ± 0.5	3.4 ± 0.1	2.5 ± 0.3	14.4 ± 2.9
	0.4	5.1 ± 0.5	3.1 ± 0.4	2.8 ± 0.2	8.3 ± 2.3	6.9 ± 0.5	3.4 ± 0.1	2.2 ± 0.1	20.8 ± 3.6
2O31	1	3.8 ± 0.2	3.9 ± 0.3	1.8 ± 0.2	1.0 ± 0.1	2.6 ± 0.1	2.5 ± 0.1	1.3 ± 0.1	2.0 ± 0.3
	0.9	3.5 ± 0.3	3.6 ± 0.4	1.9 ± 0.1	0.8 ± 0.0	2.8 ± 0.1	2.2 ± 0.1	1.6 ± 0.1	2.5 ± 0.2
	0.8	3.5 ± 0.3	3.5 ± 0.3	1.7 ± 0.0	1.2 ± 0.2	2.7 ± 0.1	3.0 ± 0.3	1.4 ± 0.1	1.7 ± 0.1
	0.7	3.2 ± 0.2	3.2 ± 0.2	1.6 ± 0.0	0.9 ± 0.1	2.5 ± 0.0	2.9 ± 0.2	1.3 ± 0.0	1.4 ± 0.1
	0.6	3.7 ± 0.5	3.5 ± 0.3	1.9 ± 0.3	2.0 ± 1.4	2.4 ± 0.2	2.3 ± 0.2	1.3 ± 0.1	1.6 ± 0.5
	0.5	5.1 ± 0.2	3.6 ± 0.1	2.3 ± 0.1	7.7 ± 1.1	4.9 ± 0.3	2.9 ± 0.2	1.4 ± 0.1	10.2 ± 1.4
	0.4	5.4 ± 0.4	3.8 ± 0.4	2.3 ± 0.1	8.5 ± 1.0	5.6 ± 0.4	3.1 ± 0.2	1.3 ± 0.1	14.0 ± 1.9

Table S4. (continued)

PDB	γ	construct 1				construct 2			
		All	UA subunit	CG subunit	Interface	All	UA subunit	CG subunit	Interface
1XX8	1	4.5 ± 0.3	4.0 ± 0.5	3.6 ± 0.1	2.5 ± 0.5	5.4 ± 0.2	5.8 ± 0.2	2.5 ± 0.2	4.8 ± 0.5
	0.9	4.1 ± 0.4	3.8 ± 0.4	3.3 ± 0.4	1.9 ± 0.5	4.8 ± 0.3	5.0 ± 0.4	2.4 ± 0.2	4.2 ± 0.8
	0.8	3.3 ± 0.1	2.8 ± 0.2	2.8 ± 0.2	1.3 ± 0.3	5.2 ± 0.3	5.7 ± 0.2	2.3 ± 0.1	4.5 ± 0.9
	0.7	2.9 ± 0.0	2.4 ± 0.1	2.6 ± 0.1	0.9 ± 0.1	3.8 ± 0.0	4.0 ± 0.1	1.9 ± 0.1	2.7 ± 0.2
	0.6	2.7 ± 0.3	1.8 ± 0.1	2.6 ± 0.4	1.4 ± 0.5	3.6 ± 0.2	3.4 ± 0.1	1.9 ± 0.1	2.8 ± 0.8
	0.5	2.8 ± 0.1	1.7 ± 0.0	2.7 ± 0.1	1.6 ± 0.2	4.2 ± 0.4	4.0 ± 0.2	1.9 ± 0.1	4.4 ± 1.1
	0.4	3.7 ± 0.2	2.0 ± 0.2	3.4 ± 0.3	3.0 ± 0.5	4.6 ± 0.6	4.4 ± 0.3	2.0 ± 0.1	5.5 ± 1.9
1TQG	1	5.2 ± 0.1	5.9 ± 0.2	1.8 ± 0.2	6.7 ± 0.7	6.0 ± 0.6	5.4 ± 0.7	3.1 ± 0.3	6.3 ± 1.5
	0.9	5.2 ± 0.3	6.5 ± 0.6	1.8 ± 0.1	5.5 ± 0.5	4.9 ± 0.4	4.6 ± 0.4	3.2 ± 0.4	3.2 ± 0.6
	0.8	4.9 ± 0.2	5.8 ± 0.2	1.6 ± 0.1	5.5 ± 0.8	4.3 ± 0.4	3.6 ± 0.5	3.4 ± 0.4	2.9 ± 0.3
	0.7	3.5 ± 0.2	4.4 ± 0.5	1.6 ± 0.1	2.7 ± 0.2	3.5 ± 0.3	2.9 ± 0.3	2.9 ± 0.4	2.0 ± 0.3
	0.6	4.6 ± 0.7	5.1 ± 0.7	2.0 ± 0.2	5.6 ± 2.5	4.0 ± 0.4	3.6 ± 0.4	2.2 ± 0.1	3.0 ± 0.9
	0.5	14.9 ± 1.7	8.6 ± 1.0	2.6 ± 0.4	100.0 ± 23.6	17.2 ± 0.4	4.1 ± 0.3	2.3 ± 0.1	141.2 ± 7.0
	0.4	16.3 ± 0.9	7.1 ± 0.8	2.5 ± 0.2	123.5 ± 16.0	17.5 ± 0.9	3.8 ± 0.2	2.3 ± 0.1	148.7 ± 15.9
1R69	1	5.7 ± 0.4	4.5 ± 0.3	2.3 ± 0.2	8.9 ± 1.6	4.5 ± 0.2	4.8 ± 0.2	1.5 ± 0.1	4.6 ± 0.6
	0.9	5.6 ± 0.2	4.2 ± 0.2	2.1 ± 0.1	9.7 ± 1.3	3.8 ± 0.2	4.1 ± 0.2	1.3 ± 0.0	3.4 ± 0.5
	0.8	4.3 ± 0.7	3.5 ± 0.4	1.7 ± 0.3	5.7 ± 2.4	3.1 ± 0.1	3.3 ± 0.2	1.3 ± 0.1	2.1 ± 0.3
	0.7	2.7 ± 0.3	2.4 ± 0.3	1.4 ± 0.1	1.6 ± 0.7	3.1 ± 0.3	3.2 ± 0.2	1.3 ± 0.1	2.2 ± 0.5
	0.6	4.5 ± 0.7	2.8 ± 0.2	1.4 ± 0.2	8.1 ± 2.9	3.4 ± 0.2	3.6 ± 0.2	1.5 ± 0.2	2.4 ± 0.7
	0.5	8.1 ± 0.9	2.4 ± 0.1	1.7 ± 0.3	31.8 ± 7.0	9.4 ± 0.6	4.2 ± 0.5	1.4 ± 0.3	40.4 ± 6.3
	0.4	9.1 ± 0.1	2.4 ± 0.1	1.8 ± 0.3	39.2 ± 1.1	9.2 ± 0.3	4.0 ± 0.3	1.3 ± 0.2	38.6 ± 2.5
2CKX	1	3.3 ± 0.3	3.3 ± 0.4	2.3 ± 0.1	1.8 ± 0.5	5.7 ± 0.5	5.6 ± 0.4	2.1 ± 0.2	6.6 ± 1.7
	0.9	3.8 ± 0.2	4.0 ± 0.2	1.8 ± 0.1	2.8 ± 0.6	4.9 ± 0.2	4.9 ± 0.3	1.7 ± 0.1	4.6 ± 0.2
	0.8	3.0 ± 0.4	3.1 ± 0.6	1.8 ± 0.1	1.6 ± 0.4	5.1 ± 0.5	5.1 ± 0.5	1.9 ± 0.3	4.9 ± 1.1
	0.7	3.2 ± 0.2	3.1 ± 0.5	1.8 ± 0.1	1.9 ± 0.0	3.5 ± 0.1	3.9 ± 0.2	1.8 ± 0.2	1.5 ± 0.2
	0.6	4.2 ± 0.9	3.0 ± 0.5	2.2 ± 0.5	6.6 ± 3.2	3.8 ± 0.3	3.9 ± 0.2	1.8 ± 0.2	2.4 ± 0.8
	0.5	8.4 ± 1.0	4.0 ± 0.3	3.5 ± 0.1	29.5 ± 7.3	10.5 ± 0.8	6.1 ± 0.8	1.6 ± 0.1	44.4 ± 7.7
	0.4	8.4 ± 0.3	4.5 ± 0.2	3.3 ± 0.4	27.9 ± 3.7	11.3 ± 0.8	6.3 ± 0.3	1.5 ± 0.0	53.6 ± 9.0
1BKR	1	5.9 ± 0.6	6.2 ± 0.7	2.5 ± 0.3	5.9 ± 1.7	4.1 ± 0.2	4.1 ± 0.3	2.3 ± 0.2	3.0 ± 0.3
	0.9	5.5 ± 0.4	6.2 ± 0.5	2.0 ± 0.1	4.6 ± 1.1	3.8 ± 0.2	3.8 ± 0.3	1.9 ± 0.2	2.8 ± 0.4
	0.8	3.6 ± 0.4	4.0 ± 0.5	1.7 ± 0.1	1.6 ± 0.3	3.1 ± 0.2	3.3 ± 0.3	1.7 ± 0.2	1.2 ± 0.2
	0.7	3.2 ± 0.3	3.5 ± 0.4	1.6 ± 0.1	1.5 ± 0.2	2.8 ± 0.3	3.0 ± 0.3	1.4 ± 0.1	0.8 ± 0.2
	0.6	4.2 ± 0.3	4.7 ± 0.5	1.5 ± 0.1	2.7 ± 0.3	2.8 ± 0.4	2.9 ± 0.4	1.7 ± 0.3	1.2 ± 0.7
	0.5	9.3 ± 1.5	6.0 ± 0.9	1.8 ± 0.2	36.2 ± 11.2	9.0 ± 1.5	6.4 ± 1.4	1.7 ± 0.1	31.8 ± 8.4
	0.4	10.7 ± 0.7	6.8 ± 0.4	2.1 ± 0.4	45.2 ± 7.9	9.6 ± 0.6	6.0 ± 0.8	1.8 ± 0.1	36.5 ± 4.6
2A3D[e]	1	3.4 ± 0.1	3.1 ± 0.1	1.8 ± 0.2	2.6 ± 0.1	6.7 ± 0.5	6.3 ± 0.6	2.1 ± 0.4	3.7 ± 0.2
	0.9	3.2 ± 0.1	3.3 ± 0.5	1.4 ± 0.1	2.4 ± 0.3	6.1 ± 0.2	5.8 ± 0.3	1.9 ± 0.4	3.1 ± 0.3

Table S4. (continued)

PDB	γ	construct 1				construct 2			
		All	UA subunit	CG subunit	Interface	All	UA subunit	CG subunit	Interface
2A3D[e]	0.8	2.9 ± 0.1	2.7 ± 0.2	1.6 ± 0.1	2.2 ± 0.2	5.4 ± 0.4	5.1 ± 0.4	1.8 ± 0.4	2.8 ± 0.3
	0.7	2.6 ± 0.2	2.5 ± 0.3	1.4 ± 0.1	1.9 ± 0.2	4.0 ± 0.3	3.7 ± 0.2	1.7 ± 0.3	2.0 ± 0.4
	0.6	3.2 ± 0.2	2.6 ± 0.3	1.6 ± 0.0	2.5 ± 0.2	8.3 ± 0.7	4.3 ± 0.3	1.7 ± 0.1	7.3 ± 0.7
	0.5	9.3 ± 0.4	4.2 ± 0.4	1.6 ± 0.0	8.9 ± 0.3	11.8 ± 1.1	4.0 ± 0.2	1.7 ± 0.0	11.1 ± 1.2
	0.4	12.4 ± 0.2	3.0 ± 0.5	1.6 ± 0.0	12.2 ± 0.3	10.7 ± 1.5	3.6 ± 0.5	1.7 ± 0.3	10.0 ± 1.6
3GB1	1	4.1 ± 0.5	3.9 ± 0.5	1.6 ± 0.2	2.5 ± 0.4	3.9 ± 0.3	3.7 ± 0.3	2.4 ± 0.3	2.8 ± 0.2
	0.9	3.0 ± 0.7	3.0 ± 0.7	1.1 ± 0.1	1.7 ± 0.6	4.4 ± 0.3	3.7 ± 0.3	2.7 ± 0.2	3.3 ± 0.3
	0.8	2.1 ± 0.1	2.2 ± 0.1	0.9 ± 0.0	1.1 ± 0.1	4.1 ± 0.2	3.6 ± 0.3	2.4 ± 0.1	3.0 ± 0.2
	0.7	1.7 ± 0.1	1.6 ± 0.1	1.0 ± 0.1	0.9 ± 0.1	2.8 ± 0.2	2.3 ± 0.4	1.9 ± 0.1	2.1 ± 0.1
	0.6	1.9 ± 0.0	1.7 ± 0.0	1.2 ± 0.0	1.1 ± 0.1	2.7 ± 0.1	1.7 ± 0.0	2.6 ± 0.3	1.9 ± 0.0
	0.5	2.2 ± 0.0	1.9 ± 0.0	1.3 ± 0.1	1.3 ± 0.0	2.8 ± 0.3	1.6 ± 0.3	2.2 ± 0.3	2.2 ± 0.2
	0.4	2.5 ± 0.0	1.9 ± 0.1	1.4 ± 0.0	1.8 ± 0.1	2.8 ± 0.2	1.4 ± 0.1	2.2 ± 0.3	2.3 ± 0.1
1UBQ	1	3.2 ± 0.1	3.3 ± 0.2	1.2 ± 0.2	1.6 ± 0.2	3.7 ± 0.1	3.3 ± 0.3	3.0 ± 0.2	2.8 ± 0.1
	0.9	3.1 ± 0.2	3.1 ± 0.3	1.2 ± 0.1	1.6 ± 0.2	3.2 ± 0.1	2.5 ± 0.2	2.6 ± 0.2	2.5 ± 0.0
	0.8	2.8 ± 0.1	2.8 ± 0.2	1.1 ± 0.1	1.5 ± 0.1	3.1 ± 0.1	2.5 ± 0.1	2.6 ± 0.2	2.4 ± 0.1
	0.7	2.5 ± 0.0	2.5 ± 0.0	0.9 ± 0.0	1.2 ± 0.0	2.9 ± 0.1	2.4 ± 0.2	2.4 ± 0.1	2.2 ± 0.0
	0.6	2.6 ± 0.2	2.4 ± 0.1	1.8 ± 0.3	1.4 ± 0.4	2.9 ± 0.1	1.9 ± 0.2	3.0 ± 0.3	2.1 ± 0.1
	0.5	2.8 ± 0.0	2.6 ± 0.1	1.5 ± 0.2	1.6 ± 0.1	3.3 ± 0.1	2.4 ± 0.1	2.7 ± 0.1	2.6 ± 0.1
	0.4	2.9 ± 0.1	2.5 ± 0.1	1.7 ± 0.2	1.8 ± 0.1	3.1 ± 0.1	2.1 ± 0.1	2.7 ± 0.1	2.4 ± 0.1
1RA4	1	1.6 ± 0.1	1.4 ± 0.2	1.5 ± 0.0	0.3 ± 0.1	3.7 ± 0.2	4.0 ± 0.2	1.6 ± 0.1	0.6 ± 0.1
	0.9	1.5 ± 0.0	1.0 ± 0.0	1.5 ± 0.0	0.1 ± 0.0	3.1 ± 0.3	3.3 ± 0.3	1.5 ± 0.1	0.5 ± 0.1
	0.8	1.4 ± 0.0	1.0 ± 0.0	1.5 ± 0.0	0.1 ± 0.0	2.2 ± 0.1	2.3 ± 0.2	1.3 ± 0.1	0.2 ± 0.0
	0.7	1.3 ± 0.0	0.9 ± 0.0	1.4 ± 0.0	0.1 ± 0.0	2.1 ± 0.1	2.3 ± 0.1	1.1 ± 0.1	0.1 ± 0.0
	0.6	1.2 ± 0.0	0.8 ± 0.0	1.3 ± 0.1	0.0 ± 0.0	1.9 ± 0.1	2.0 ± 0.2	1.0 ± 0.1	0.1 ± 0.0
	0.5	1.2 ± 0.0	0.9 ± 0.1	1.2 ± 0.0	0.1 ± 0.0	1.7 ± 0.1	1.8 ± 0.1	1.0 ± 0.0	0.1 ± 0.0
	0.4	1.1 ± 0.0	0.9 ± 0.0	1.1 ± 0.0	0.1 ± 0.0	1.7 ± 0.1	1.8 ± 0.1	1.0 ± 0.0	0.1 ± 0.0
2KRY	1	3.3 ± 0.1	2.5 ± 0.1	3.3 ± 0.2	0.9 ± 0.1	5.6 ± 0.4	6.2 ± 0.4	2.8 ± 0.3	3.3 ± 0.8
	0.9	2.5 ± 0.1	1.5 ± 0.1	2.8 ± 0.1	0.4 ± 0.0	5.6 ± 0.3	6.2 ± 0.4	2.4 ± 0.2	3.6 ± 0.2
	0.8	2.6 ± 0.1	1.9 ± 0.2	2.7 ± 0.1	0.4 ± 0.1	4.6 ± 0.2	5.2 ± 0.2	2.1 ± 0.1	2.0 ± 0.4
	0.7	1.9 ± 0.1	1.5 ± 0.2	1.9 ± 0.1	0.3 ± 0.1	3.6 ± 0.3	4.2 ± 0.4	1.5 ± 0.1	1.2 ± 0.5
	0.6	1.8 ± 0.0	1.6 ± 0.1	1.7 ± 0.0	0.2 ± 0.0	3.1 ± 0.2	3.5 ± 0.3	1.5 ± 0.1	0.9 ± 0.1
	0.5	1.6 ± 0.1	1.4 ± 0.1	1.6 ± 0.1	0.2 ± 0.0	5.9 ± 1.4	6.7 ± 1.6	1.4 ± 0.1	5.2 ± 2.5
	0.4	2.0 ± 0.1	1.5 ± 0.1	2.0 ± 0.1	0.4 ± 0.0	5.9 ± 1.3	6.8 ± 1.5	1.4 ± 0.1	4.5 ± 2.2
2VIM	1	3.3 ± 0.1	3.2 ± 0.2	3.1 ± 0.2	0.5 ± 0.1	3.7 ± 0.2	4.2 ± 0.2	2.5 ± 0.1	0.8 ± 0.1
	0.9	3.3 ± 0.4	3.0 ± 0.4	3.3 ± 0.3	0.6 ± 0.3	3.0 ± 0.4	3.4 ± 0.4	2.0 ± 0.3	0.7 ± 0.1
	0.8	3.0 ± 0.2	2.6 ± 0.3	3.0 ± 0.2	0.3 ± 0.1	2.4 ± 0.1	2.6 ± 0.0	1.8 ± 0.1	0.4 ± 0.1
	0.7	2.4 ± 0.2	2.1 ± 0.3	2.2 ± 0.2	0.6 ± 0.1	2.3 ± 0.1	2.5 ± 0.2	1.6 ± 0.0	0.5 ± 0.0

Table S4. (continued)

PDB	γ	construct 1				construct 2			
		All	UA subunit	CG subunit	Interface	All	UA subunit	CG subunit	Interface
2VIM	0.6	2.2 ± 0.2	1.9 ± 0.2	2.2 ± 0.2	0.4 ± 0.0	1.8 ± 0.1	1.9 ± 0.1	1.6 ± 0.1	0.2 ± 0.0
	0.5	2.3 ± 0.1	2.1 ± 0.1	2.1 ± 0.1	0.3 ± 0.0	1.8 ± 0.0	1.9 ± 0.1	1.4 ± 0.0	0.2 ± 0.0
	0.4	2.2 ± 0.1	2.0 ± 0.0	2.2 ± 0.1	0.2 ± 0.0	1.9 ± 0.0	2.1 ± 0.0	1.4 ± 0.0	0.3 ± 0.0
1CRN	1	3.8 ± 0.2	2.7 ± 0.1	1.0 ± 0.1	5.1 ± 0.7	3.9 ± 0.6	3.0 ± 0.3	1.5 ± 0.1	5.2 ± 2.2
	0.9	3.7 ± 0.2	3.0 ± 0.2	1.0 ± 0.0	4.4 ± 0.7	3.5 ± 0.2	2.9 ± 0.2	1.4 ± 0.1	3.4 ± 0.6
	0.8	2.9 ± 0.5	2.6 ± 0.4	1.0 ± 0.0	2.6 ± 1.3	3.1 ± 0.1	2.7 ± 0.2	1.4 ± 0.0	2.5 ± 0.2
	0.7	2.2 ± 0.1	2.5 ± 0.2	1.0 ± 0.0	0.6 ± 0.1	2.3 ± 0.2	1.5 ± 0.2	1.3 ± 0.0	1.6 ± 0.4
	0.6	2.2 ± 0.0	2.4 ± 0.0	1.0 ± 0.0	0.9 ± 0.1	3.0 ± 0.2	1.8 ± 0.2	1.4 ± 0.0	3.2 ± 0.5
	0.5	2.2 ± 0.1	2.4 ± 0.0	0.9 ± 0.0	0.9 ± 0.2	3.3 ± 0.1	1.7 ± 0.1	1.4 ± 0.0	4.1 ± 0.4
	0.4	2.4 ± 0.2	2.5 ± 0.0	0.9 ± 0.0	1.4 ± 0.4	3.3 ± 0.2	1.8 ± 0.2	1.4 ± 0.0	4.2 ± 0.5
1OCR[e]	1	3.6 ± 0.3	3.0 ± 0.1	1.8 ± 0.1	3.5 ± 0.9	6.3 ± 0.5	6.1 ± 0.6	3.0 ± 0.3	7.5 ± 1.3
	0.9	3.6 ± 0.2	3.2 ± 0.2	1.7 ± 0.1	3.5 ± 0.8	6.2 ± 0.4	5.9 ± 0.4	3.4 ± 0.3	7.1 ± 0.6
	0.8	3.1 ± 0.3	2.9 ± 0.2	1.5 ± 0.1	2.6 ± 0.7	5.0 ± 0.5	4.2 ± 0.4	2.7 ± 0.2	6.2 ± 1.4
	0.7	2.9 ± 0.2	3.1 ± 0.2	1.5 ± 0.2	1.4 ± 0.5	4.5 ± 0.6	3.3 ± 0.4	2.8 ± 0.2	5.6 ± 2.1
	0.6	3.3 ± 0.2	3.5 ± 0.1	2.0 ± 0.2	1.5 ± 0.3	5.0 ± 0.5	3.1 ± 0.2	3.2 ± 0.2	7.9 ± 2.4
	0.5	6.3 ± 0.4	3.4 ± 0.1	2.2 ± 0.2	16.2 ± 2.4	8.1 ± 0.2	2.5 ± 0.2	2.9 ± 0.3	28.9 ± 1.7
	0.4	7.2 ± 0.5	3.5 ± 0.1	2.2 ± 0.1	22.0 ± 4.0	8.9 ± 0.6	2.8 ± 0.5	2.8 ± 0.2	35.9 ± 4.6
1PGB	1	5.5 ± 0.5	5.0 ± 0.5	1.6 ± 0.1	6.5 ± 1.1	4.2 ± 0.5	4.0 ± 0.5	2.4 ± 0.2	4.5 ± 1.2
	0.9	4.1 ± 1.0	3.8 ± 0.9	1.4 ± 0.2	4.2 ± 1.7	3.8 ± 0.3	3.4 ± 0.4	2.7 ± 0.4	2.7 ± 0.5
	0.8	3.3 ± 0.3	3.0 ± 0.4	1.2 ± 0.0	2.4 ± 0.5	3.7 ± 0.2	3.1 ± 0.2	2.8 ± 0.4	2.6 ± 0.3
	0.7	1.6 ± 0.1	1.4 ± 0.1	1.1 ± 0.0	0.4 ± 0.1	2.9 ± 0.1	2.0 ± 0.3	2.5 ± 0.2	1.4 ± 0.2
	0.6	2.2 ± 0.3	2.1 ± 0.3	1.3 ± 0.1	0.8 ± 0.2	2.3 ± 0.1	1.3 ± 0.1	2.1 ± 0.1	0.9 ± 0.1
	0.5	2.5 ± 0.3	1.9 ± 0.4	1.6 ± 0.2	1.4 ± 0.3	2.3 ± 0.2	1.6 ± 0.2	1.9 ± 0.1	1.1 ± 0.2
	0.4	2.5 ± 0.1	1.8 ± 0.1	1.6 ± 0.1	1.6 ± 0.1	2.7 ± 0.3	1.8 ± 0.2	2.1 ± 0.2	1.6 ± 0.4
2IUG	1	4.1 ± 0.1	3.6 ± 0.2	3.9 ± 0.1	1.4 ± 0.3	4.9 ± 0.5	5.9 ± 0.6	2.3 ± 0.1	1.8 ± 0.5
	0.9	3.2 ± 0.2	2.6 ± 0.2	3.4 ± 0.3	0.7 ± 0.2	4.7 ± 0.1	5.4 ± 0.1	2.5 ± 0.3	1.9 ± 0.3
	0.8	3.0 ± 0.2	2.4 ± 0.2	2.9 ± 0.2	0.9 ± 0.2	4.5 ± 0.2	5.2 ± 0.3	2.3 ± 0.1	1.8 ± 0.4
	0.7	2.6 ± 0.1	2.1 ± 0.1	2.5 ± 0.1	0.8 ± 0.2	3.8 ± 0.1	4.4 ± 0.1	2.3 ± 0.2	1.1 ± 0.1
	0.6	2.3 ± 0.1	1.9 ± 0.1	2.3 ± 0.1	0.5 ± 0.1	3.5 ± 0.5	4.1 ± 0.7	2.1 ± 0.2	1.0 ± 0.2
	0.5	2.3 ± 0.2	2.1 ± 0.2	2.0 ± 0.0	0.6 ± 0.2	4.6 ± 0.4	5.4 ± 0.6	2.2 ± 0.2	1.6 ± 0.3
	0.4	3.1 ± 0.2	2.7 ± 0.2	2.3 ± 0.1	1.7 ± 0.3	4.9 ± 0.3	5.6 ± 0.5	2.4 ± 0.4	2.4 ± 0.3
2QYO	1	3.6 ± 0.2	3.7 ± 0.2	1.8 ± 0.1	0.4 ± 0.0	4.3 ± 0.3	3.8 ± 0.4	4.3 ± 0.3	0.2 ± 0.0
	0.9	3.4 ± 0.2	3.5 ± 0.2	1.8 ± 0.1	0.3 ± 0.1	3.5 ± 0.1	2.9 ± 0.1	3.7 ± 0.1	0.1 ± 0.0
	0.8	3.4 ± 0.4	3.5 ± 0.4	1.8 ± 0.1	0.3 ± 0.1	4.3 ± 0.3	2.8 ± 0.2	4.5 ± 0.4	0.1 ± 0.1
	0.7	3.3 ± 0.4	3.5 ± 0.4	1.7 ± 0.1	0.3 ± 0.1	4.2 ± 0.5	2.6 ± 0.3	4.5 ± 0.5	0.1 ± 0.1
	0.6	3.9 ± 0.3	4.1 ± 0.3	1.7 ± 0.1	0.4 ± 0.1	4.4 ± 0.3	3.2 ± 0.3	4.6 ± 0.3	0.2 ± 0.1
	0.5	5.0 ± 0.3	5.2 ± 0.4	2.1 ± 0.2	1.0 ± 0.1	4.4 ± 0.2	2.8 ± 0.2	4.6 ± 0.3	0.3 ± 0.1

Table S4. (continued)

PDB	γ	construct 1				construct 2			
		All	UA subunit	CG subunit	Interface	All	UA subunit	CG subunit	Interface
2QYO	0.4	5.8 ± 0.4	5.8 ± 0.5	2.3 ± 0.2	2.6 ± 0.8	4.7 ± 0.4	3.3 ± 0.2	4.9 ± 0.4	0.3 ± 0.1
1OPS	1	3.3 ± 0.4	3.3 ± 0.4	2.1 ± 0.2	1.0 ± 0.3	3.1 ± 0.2	3.2 ± 0.4	2.3 ± 0.2	1.5 ± 0.2
	0.9	3.3 ± 0.2	3.2 ± 0.3	1.9 ± 0.0	1.1 ± 0.2	2.8 ± 0.2	2.6 ± 0.2	2.1 ± 0.1	1.4 ± 0.3
	0.8	2.8 ± 0.2	2.8 ± 0.2	1.6 ± 0.1	0.7 ± 0.2	2.3 ± 0.2	2.2 ± 0.2	1.7 ± 0.1	1.0 ± 0.3
	0.7	2.7 ± 0.2	2.2 ± 0.1	1.9 ± 0.1	1.0 ± 0.4	2.1 ± 0.1	1.8 ± 0.0	1.6 ± 0.1	0.7 ± 0.1
	0.6	2.2 ± 0.1	2.0 ± 0.1	1.7 ± 0.1	0.6 ± 0.1	2.0 ± 0.2	2.1 ± 0.3	1.5 ± 0.1	0.5 ± 0.2
	0.5	2.0 ± 0.0	1.8 ± 0.0	1.6 ± 0.1	0.4 ± 0.0	2.1 ± 0.1	2.1 ± 0.1	1.7 ± 0.2	0.5 ± 0.1
	0.4	2.5 ± 0.1	2.2 ± 0.1	2.0 ± 0.0	0.9 ± 0.1	3.2 ± 0.2	3.3 ± 0.2	2.1 ± 0.2	2.2 ± 0.5

a) all the BB sites and the Cα sites were used for RMSD calculation. b) In each case, the averages and the standard deviations were obtained over three independent 50-ns simulations, the second halves of which were employed for the analysis. c) Each protein was associated with two PACEm constructs. d) The error indicates the standard errors of the mean. e) shown in red are the RMSD data for the constructs that did not exhibit a mean RMSD value below 3.5 Å at any γ value examined.

Table S5. The average RMSD (Å) of the backbone (BB/Cα sites) for dimeric complexes using PACEm with different choices of scaling factor γ. [a, b, c]

γ	PDB	All	UA subunit	CG subunit	Interface	PDB	All	UA subunit	CG subunit	Interface
1.0	4C4K	5.3 ± 1.4	4.2 ± 0.3	1.3 ± 0.0	4.0 ± 1.7	6GB2	5.9 ± 2.5	2.9 ± 0.2	1.1 ± 0.0	5.3 ± 2.7
0.9		4.3 ± 0.7	3.7 ± 0.2	1.2 ± 0.0	3.2 ± 0.9		7.5 ± 3.7	3.2 ± 0.1	1.1 ± 0.0	6.9 ± 3.8
0.8		3.6 ± 0.4	3.8 ± 0.6	1.3 ± 0.1	2.2 ± 0.2		4.1 ± 0.6	3.0 ± 0.2	1.2 ± 0.0	3.4 ± 0.7
0.7		4.2 ± 0.4	3.9 ± 0.3	1.3 ± 0.1	3.0 ± 0.4		3.9 ± 0.4	3.3 ± 0.2	1.1 ± 0.0	2.9 ± 0.6
0.6		9.5 ± 3.6	3.5 ± 0.2	1.3 ± 0.0	8.8 ± 3.8		11.9 ± 3.4	3.3 ± 0.2	1.1 ± 0.0	11.5 ± 3.5
0.5		17.8 ± 1.6	3.9 ± 0.3	1.3 ± 0.0	17.6 ± 1.7		17.8 ± 2.7	3.0 ± 0.1	1.2 ± 0.0	17.7 ± 2.8
0.4		20.8 ± 2.9	4.0 ± 0.2	1.3 ± 0.0	20.6 ± 2.9		19.0 ± 3.4	2.9 ± 0.1	1.1 ± 0.0	18.9 ± 3.5
1.0	2VLN	4.4 ± 0.7	3.6 ± 0.3	1.4 ± 0.1	3.6 ± 0.8	4G0N	3.9 ± 0.3	3.7 ± 0.4	1.3 ± 0.1	3.2 ± 0.3
0.9		7.2 ± 3.8	3.8 ± 0.4	1.4 ± 0.1	6.5 ± 3.9		3.6 ± 0.1	2.9 ± 0.2	1.2 ± 0.0	3.1 ± 0.2
0.8		4.0 ± 0.1	3.6 ± 0.1	1.3 ± 0.0	3.2 ± 0.2		2.8 ± 0.2	2.4 ± 0.0	1.3 ± 0.0	2.3 ± 0.3
0.7		3.9 ± 0.1	3.5 ± 0.3	1.4 ± 0.1	3.3 ± 0.0		2.9 ± 0.2	2.0 ± 0.2	1.3 ± 0.1	2.6 ± 0.2
0.6		12.2 ± 2.9	3.6 ± 0.3	1.3 ± 0.0	11.9 ± 2.9		7.0 ± 1.3	2.2 ± 0.1	1.4 ± 0.0	6.8 ± 1.3
0.5		17.7 ± 0.8	4.2 ± 0.3	1.3 ± 0.0	17.5 ± 0.8		20.0 ± 3.2	2.1 ± 0.1	1.4 ± 0.1	20.0 ± 3.2
0.4		19.9 ± 1.8	3.8 ± 0.2	1.3 ± 0.0	19.7 ± 1.9		21.0 ± 2.3	2.2 ± 0.1	1.3 ± 0.1	20.9 ± 2.3
1.0	1VET	3.3 ± 0.2	3.5 ± 0.2	1.8 ± 0.1	1.7 ± 0.2	1BRS[d]	5.3 ± 0.2	5.6 ± 0.3	1.3 ± 0.1	3.7 ± 0.2
0.9		3.5 ± 0.3	4.0 ± 0.4	1.6 ± 0.0	1.7 ± 0.2		5.4 ± 1.3	3.9 ± 0.3	1.4 ± 0.2	4.4 ± 1.5
0.8		3.5 ± 0.3	4.0 ± 0.3	1.6 ± 0.1	1.5 ± 0.2		5.8 ± 1.0	4.0 ± 0.4	1.4 ± 0.0	4.9 ± 1.2
0.7		3.1 ± 0.2	3.5 ± 0.2	1.4 ± 0.1	1.6 ± 0.2		4.9 ± 0.7	4.0 ± 0.4	1.2 ± 0.0	4.0 ± 0.7
0.6		3.5 ± 0.4	3.2 ± 0.2	1.7 ± 0.2	2.1 ± 0.6		12.6 ± 1.5	3.3 ± 0.2	1.3 ± 0.1	12.4 ± 1.5
0.5		24.1 ± 2.4	3.9 ± 0.1	1.6 ± 0.1	23.9 ± 2.4		25.0 ± 3.4	3.7 ± 0.3	1.3 ± 0.1	24.9 ± 3.4
0.4		23.9 ± 1.6	4.1 ± 0.2	1.6 ± 0.1	23.7 ± 1.6		26.1 ± 3.2	3.4 ± 0.3	1.5 ± 0.1	26.0 ± 3.2
1.0	4JW3	5.8 ± 0.8	5.7 ± 1.0	1.4 ± 0.1	4.0 ± 0.9	4INS	7.2 ± 2.7	3.3 ± 0.3	1.4 ± 0.1	6.4 ± 2.9
0.9		5.1 ± 0.8	5.3 ± 1.0	1.4 ± 0.1	3.3 ± 0.7		6.3 ± 1.6	3.0 ± 0.3	1.4 ± 0.1	5.6 ± 1.8
0.8		4.3 ± 0.6	4.4 ± 0.4	1.2 ± 0.0	2.9 ± 0.6		4.0 ± 0.6	3.4 ± 0.4	1.4 ± 0.0	2.9 ± 0.7
0.7		3.9 ± 0.2	3.6 ± 0.2	1.3 ± 0.0	3.2 ± 0.2		3.8 ± 0.6	2.9 ± 0.1	1.3 ± 0.0	3.0 ± 0.8
0.6		5.6 ± 1.0	3.8 ± 0.3	1.2 ± 0.0	4.8 ± 1.1		18.3 ± 4.4	2.9 ± 0.4	1.3 ± 0.0	18.1 ± 4.4
0.5		17.7 ± 1.5	3.1 ± 0.2	1.2 ± 0.0	17.5 ± 1.5		32.3 ± 3.1	3.3 ± 0.6	1.3 ± 0.0	32.2 ± 3.1
0.4		22.4 ± 1.3	3.2 ± 0.4	1.2 ± 0.0	22.3 ± 1.3		23.4 ± 2.9	3.7 ± 0.6	1.3 ± 0.0	23.2 ± 2.9
1.0	5ELU	2.6 ± 0.3	2.3 ± 0.2	1.4 ± 0.0	1.8 ± 0.4					
0.9		2.7 ± 0.0	2.7 ± 0.1	1.4 ± 0.0	1.9 ± 0.1					
0.8		2.6 ± 0.3	2.4 ± 0.3	1.3 ± 0.0	1.7 ± 0.4					
0.7		2.3 ± 0.1	2.0 ± 0.1	1.3 ± 0.1	1.3 ± 0.4					
0.6		6.6 ± 1.9	1.7 ± 0.1	1.3 ± 0.0	6.4 ± 2.0					
0.5		17.9 ± 2.6	2.3 ± 0.3	1.3 ± 0.0	17.8 ± 2.6					
0.4		23.3 ± 2.1	2.1 ± 0.2	1.3 ± 0.0	23.3 ± 2.1					

a) all the BB sites and the Cα sites were used for RMSD calculation. b) In each case, the averages and the standard deviations were obtained over three independent 50-ns simulations, the second halves of which were employed for the analysis. c) The error indicates the standard errors of the mean. d) shown in red are the RMSD data for the constructs that did not exhibit a mean RMSD value below 4.0 Å at any γ value examined.

Table S6. The average RMSD of the backbone (Cα sites) for three membrane proteins obtained from PACE simulations with various (η, ζ) pairs.[a, b, c]

Systems		PACE simulation					AA simulation
		ζ = 1.00	ζ = 1.25	ζ = 1.50	ζ = 2.00	ζ = 3.00	
ARI (5AX0)	η = 1	4.16 ± 0.57	4.22 ± 0.48	3.55 ± 0.29	3.24 ± 0.28	3.22 ± 0.115	1.83 ± 0.10
	η = 0.95	4.03 ± 0.27	3.67 ± 0.13	3.02 ± 0.02	3.30 ± 0.21	3.42 ± 0.04	
	η = 0.9	3.84 ± 0.22	4.44 ± 0.27	3.12 ± 0.14	3.08 ± 0.24	3.97 ± 0.13	
DAP12 (4WOL)	η = 1	6.28 ± 0.57	4.13 ± 0.23	4.67 ± 0.90	4.88 ± 0.64	3.86 ± 0.94	3.74 ± 0.28
	η = 0.95	6.22 ± 0.61	3.99 ± 0.28	3.78 ± 0.15	3.41 ± 0.66	3.53 ± 0.69	
	η = 0.9	6.52 ± 0.31	3.56 ± 0.35	3.39 ± 0.15	2.67 ± 0.26	3.39 ± 0.37	
KcsA (1K4C)	η = 1	4.04 ± 0.05	3.13 ± 0.04	3.01 ± 0.07	2.66 ± 0.03	3.61 ± 0.17	2.05 ± 0.25
	η = 0.95	3.52 ± 0.28	3.34 ± 0.22	2.92 ± 0.10	3.20 ± 0.14	3.37 ± 0.03	
	η = 0.9	4.05 ± 0.16	3.21 ± 0.05	3.00 ± 0.11	3.01± 0.18	3.16 ± 0.08	

a) all the Cα sites were used for RMSD calculation. b) In each case, the averages and the standard deviations were obtained over three independent 100-ns simulations, the second halves of which were employed for the analysis. c) The error indicates the standard errors of the mean.

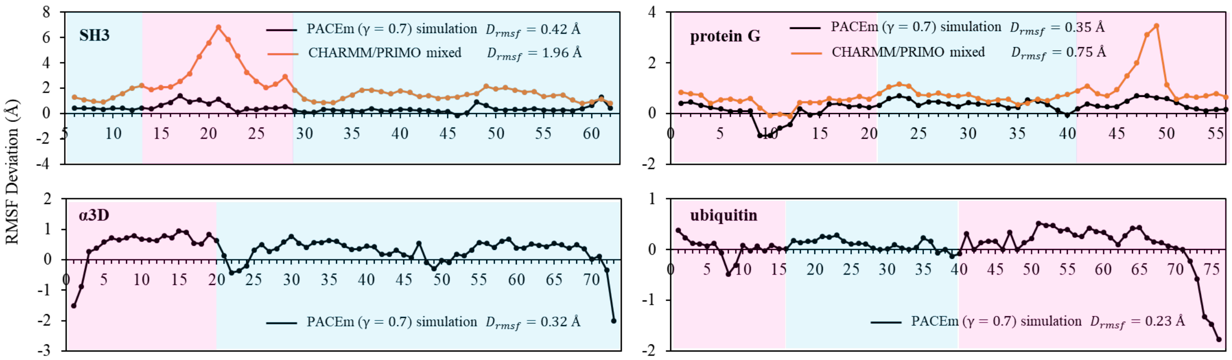

Figure S1. The average deviation of RMSF across four proteins. Magenta and cyan regions indicate the subunits modeled at UA and CG resolutions, respectively. The black curve represents the RMSF deviation between PACEm and AA simulations. The orange curve represents the RMSF deviation between CHARMM/PRIMO mixed resolution model and AA simulations, with the RMSF data for the CHARMM/PRIMO model sourced from Figures 7G and 7H in ref 1.

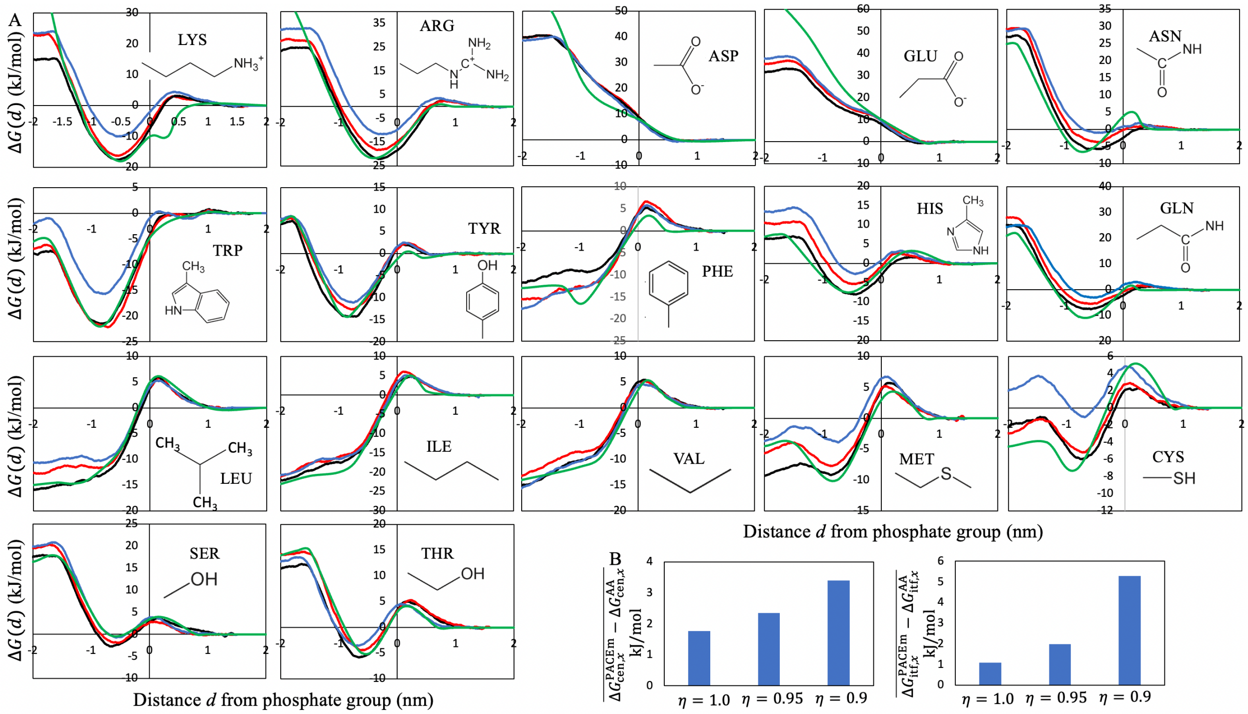

Figure S2. Free energy $\Delta G(d)$ for transferring amino acid side-chain analogues from solute to DOPC membrane. (A) PMFs of transferring amino acid side-chain analogues from solute to DOPC membrane. The results derived from the original PACE model ($\eta = 1.0$) are shown as in black curves. The results from PACEm under $\eta = 0.95$ and $\eta = 0.9$ are shown as red and blue curves, respectively. The green curves represent the PMFs obtained with the OPLS-AA forcefield with the Berger lipid model as reported in ref 2. The PMFs were calculated following the procedure detailed in ref 3. (B) The average deviation of $\Delta G_{\text{cen},x}$ (left) and $\Delta G_{\text{itf},x}$ (right) under PACEm with different η from those obtained from the AA simulations. For sidechain analogue x, $\Delta G_{\text{cen},x}$ was taken from its PMF value at $d = -2.0$ and $\Delta G_{\text{itf},x}$, when applicable, was taken from its PMF value at the minimum position near $d = 0.0$. The PMF deviation from the AA results for this analogue is thus quantified with differential quantities $\Delta G_{\text{cen},x}^{\text{PACE}} - \Delta G_{\text{cen},x}^{\text{AA}}$ and $\Delta G_{\text{itf},x}^{\text{PACE}} - \Delta G_{\text{itf},x}^{\text{AA}}$. Shown in the bar charts are the average of these quantities over all the analogues that carry no net charge (for $\Delta G_{\text{cen},x}^{\text{PACE}} - \Delta G_{\text{cen},x}^{\text{AA}}$) or exhibit a PMF minimum near $d = 0.0$ (for $\Delta G_{\text{itf},x}^{\text{PACE}} - \Delta G_{\text{itf},x}^{\text{AA}}$).

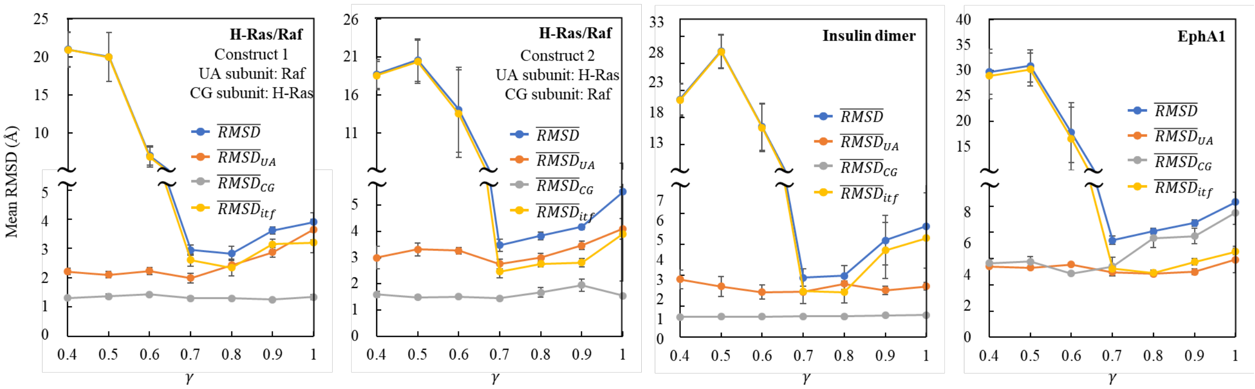

Figure S3. The variations in each type of mean RMSDs of dimeric complexes obtained with different choices of scaling factor γ.

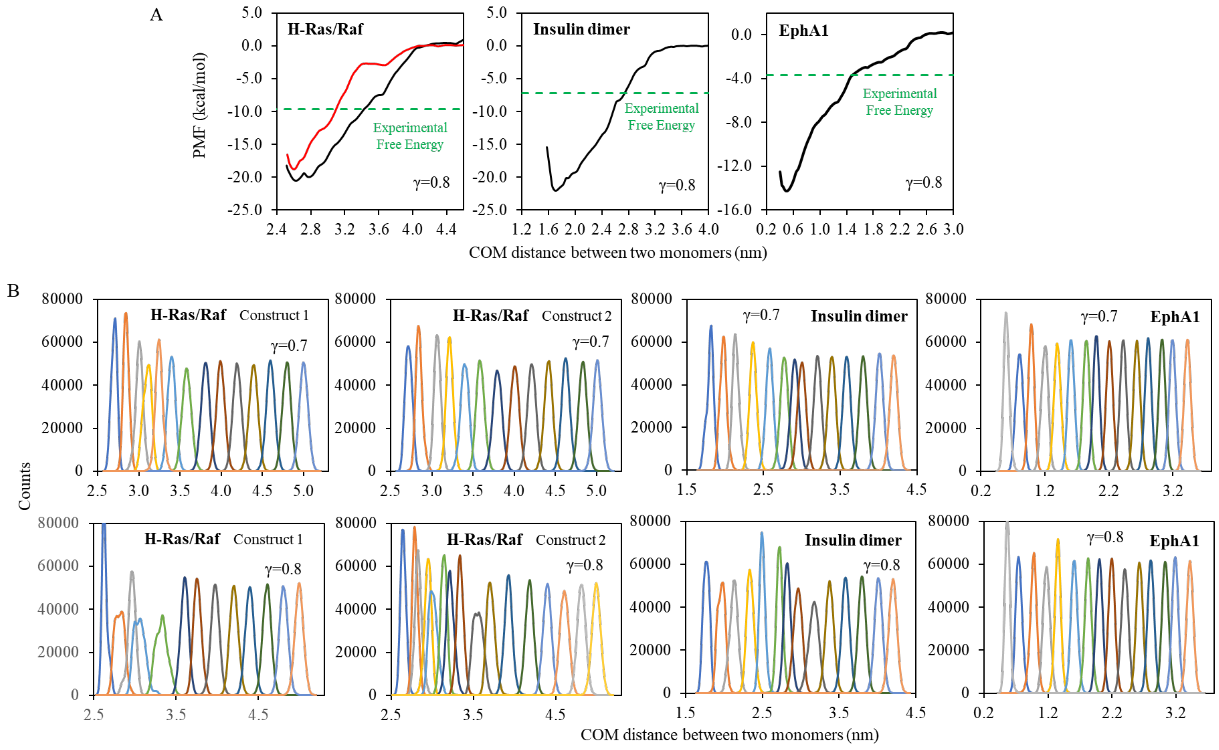

Figure S4. (A) The PMFs of complex association (black curves). The green dashed line indicates association free energy obtained from experiments. In the results for H-Ras/Raf, the black curve denotes the PMF obtained when the presentation of the complex is construct 1: H-Ras was modeled at the CG resolution and Raf was modeled at the UA resolution. The red curve denotes the PMF obtained after the representation resolutions of the two domains were swapped (construct 2). (B) The COM histograms of the configurations obtained from umbrella sampling windows. Each window for each system was simulated for 100 ns. For each construct, there was sufficient overlap between adjacent windows.

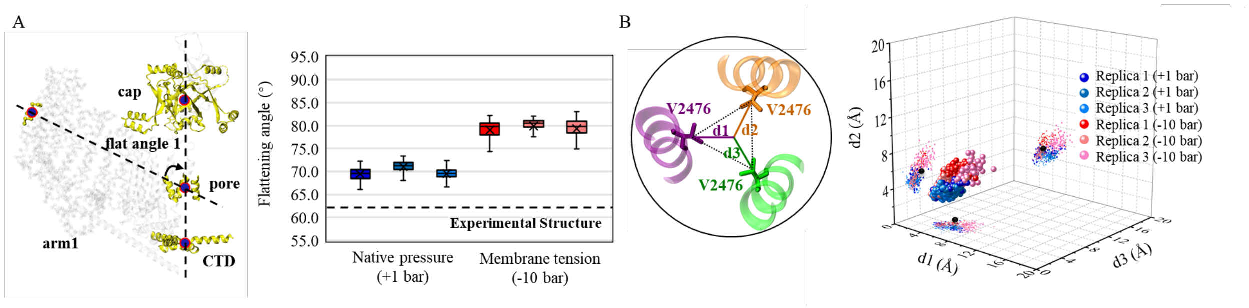

Figure S5. Piezo1 MD simulation results with ELNEDYN22 CG model. (A) The flattening angle of the three Piezo1 arms across three independent native pressure (+1 bar) simulations and three independent membrane tension (-10 bar) simulations. The angle is defined by the angle between the arm axis (determined by the COM of the outermost helix, residue 850-860, of the arm and the pore region) and the internal axis (determined by the COM of the cap and CTD region), as illustrated on the left. The values of arm 1, arm 2, and arm 3 from the final 100 ns are plotted here. The average value is indicated by the "×" symbol. (B) Definition of pore size based on pore-lining residues V2476: the average distance ($d_V = (d_1^V + d_2^V + d_3^V)/3$) of the Cβ atoms of the three valine residues to the centroid of these Cβ atoms. Panel on the right denote the plots of (d_1^V, d_2^V, d_3^V) for all the simulations. The black dots denote the distance values calculated based on the experimental structure of the closed state.

References:

[1] Kar, P.; Feig, M. Hybrid All-Atom/Coarse-Grained Simulations of Proteins by Direct Coupling of CHARMM and PRIMO Force Fields. *J. Chem. Theory Comput.* **2017**, *13* (11), 5753-5765.

[2] MacCallum, J. L.; Bennett, W. F.; Tieleman, D. P. Distribution of amino acids in a lipid bilayer from computer simulations. *Biophys. J.* **2008**, *94* (9), 3393-3404.

[3] Wan, C. K.; Han, W.; Wu, Y. D. Parameterization of PACE Force Field for Membrane Environment and Simulation of Helical Peptides and Helix-Helix Association. *J. Chem. Theory Comput.* **2012**, *8* (1), 300-313.
